# Supplementary material for: Incorporating Domain Knowledge and Structure-Based Descriptors for Machine Learning: A Case Study of Pd-Catalyzed Sonogashira Reactions
Source: Molecules. 2023 Jun 13;28(12):4730. doi: 10.3390/molecules28124730 (PMC10302643; doi:10.3390/molecules28124730)
Supplement: Supplementary file 1 [file molecules-28-04730-s001.zip › molecules-2431300-supplementary.pdf]

Supporting Information *for*

Incorporating Domain Knowledge and Structure-Based Descriptors for Machine Learning: A Case Study of Pd-Catalyzed Sonogashira Reactions

Kalok Chan, Long Thanh Ta, Yong Huang *, Haibin Su * and Zhenyang Lin *

Department of Chemistry, The Hong Kong University of Science and Technology, Clear Water Bay, Kowloon, Hong Kong SAR, China; klchanbl@connect.ust.hk (K.C.); long.ta@ust.hk (L.T.T.)

* Correspondence: yonghuang@ust.hk (Y.H.); haibinsu@ust.hk (H.S.); chzlin@ust.hk (Z.L.)

Index

1. Plots of ΔG‡(L-S) against substrate for Ligands 1-17 .... S2

2. Plots of ΔG‡(L-S) against ligand for Arylbromides 1-20 .... S11

3. Parity plots for cross validation sets 1-4 .... S21

5. Additional tables S29

1. Plots of ΔG‡(L-S) against substrate for Ligands 1-17

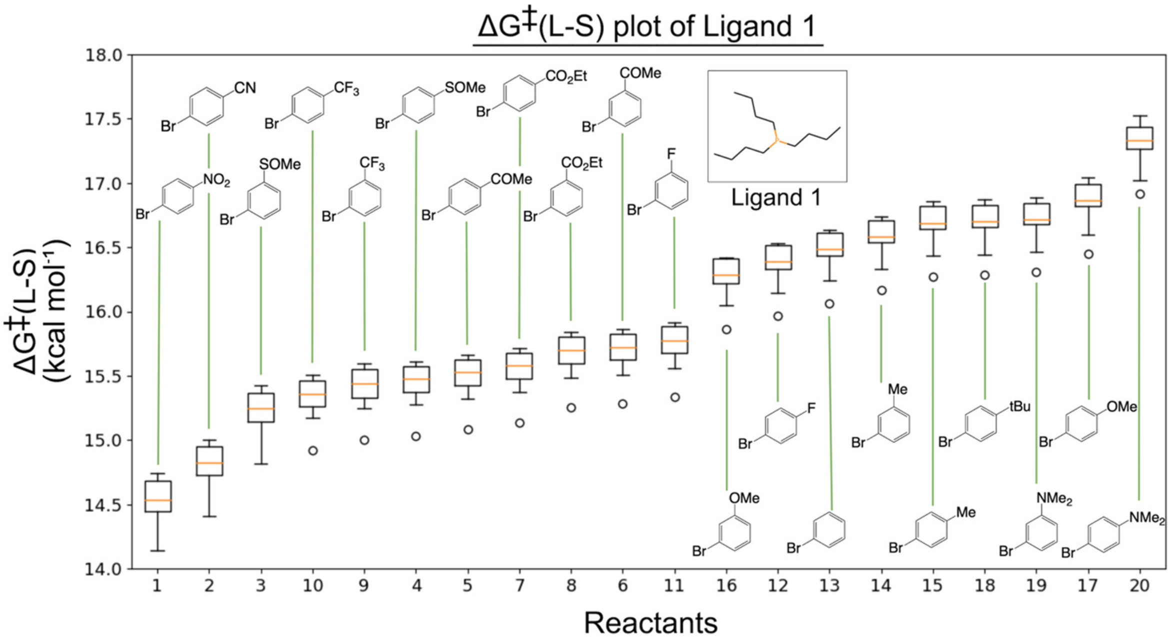

Figure S1. Plot of ΔG‡(L-S) against meta- and para-substituted arylbromide for ligand 1 (tri-n-butylphosphine).

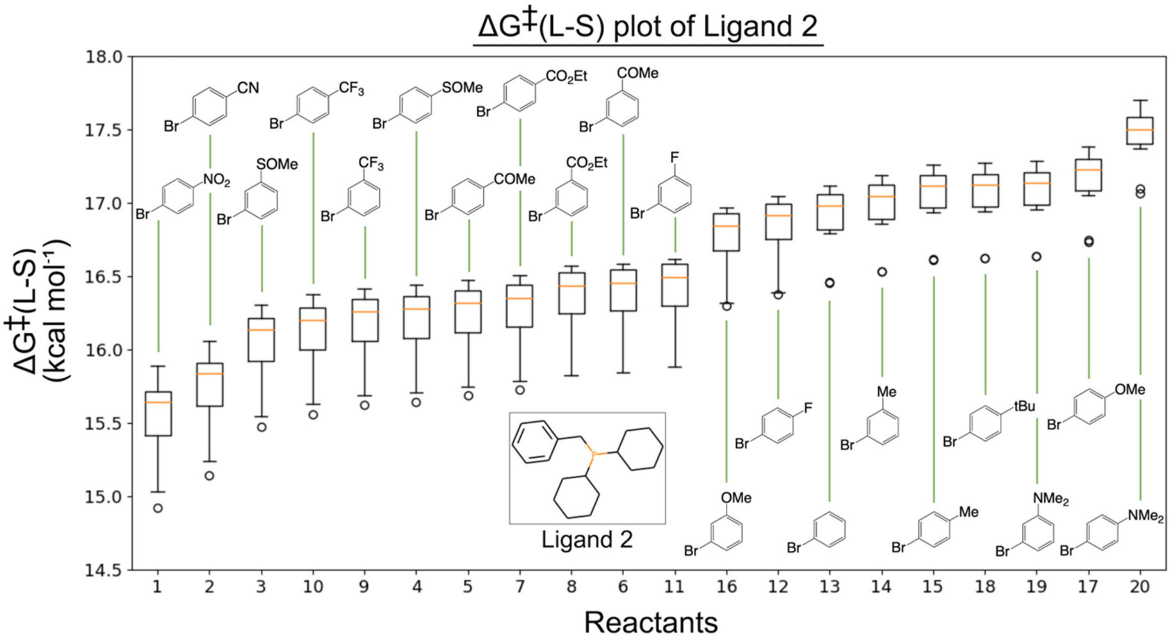

Figure S2. Plot of ΔG‡(L-S) against meta- and para-substituted arylbromide for ligand 2 (benzyl(dicyclohexyl)phosphine).

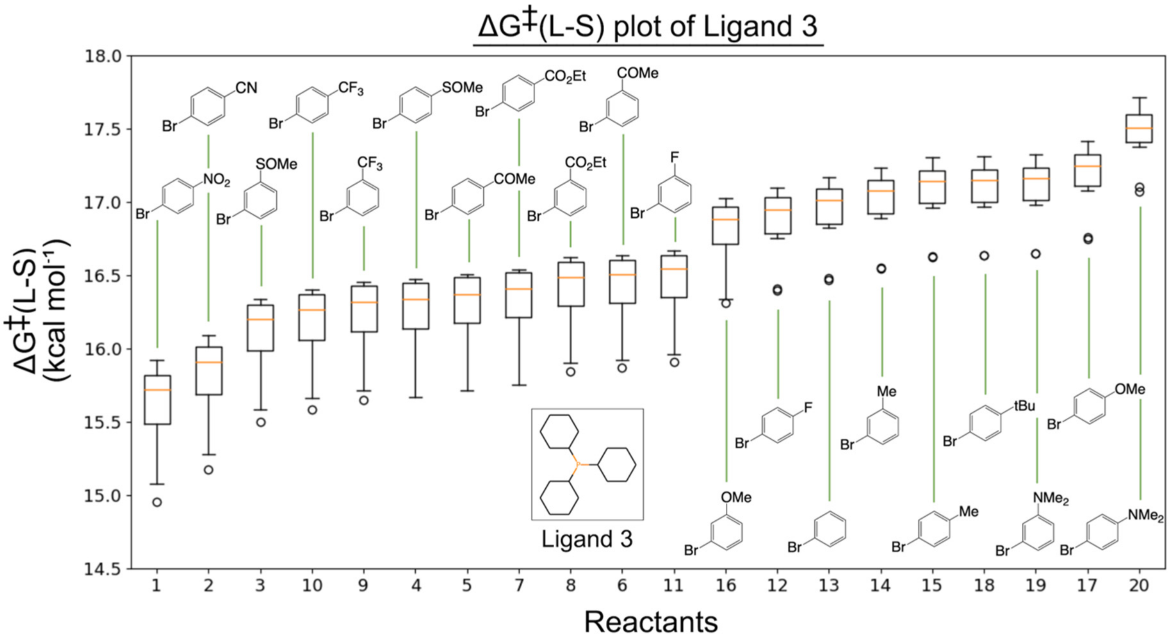

Figure S3. Plot of ΔG‡(L-S) against meta- and para-substituted arylbromide for ligand 3 (tricyclohexylphosphane).

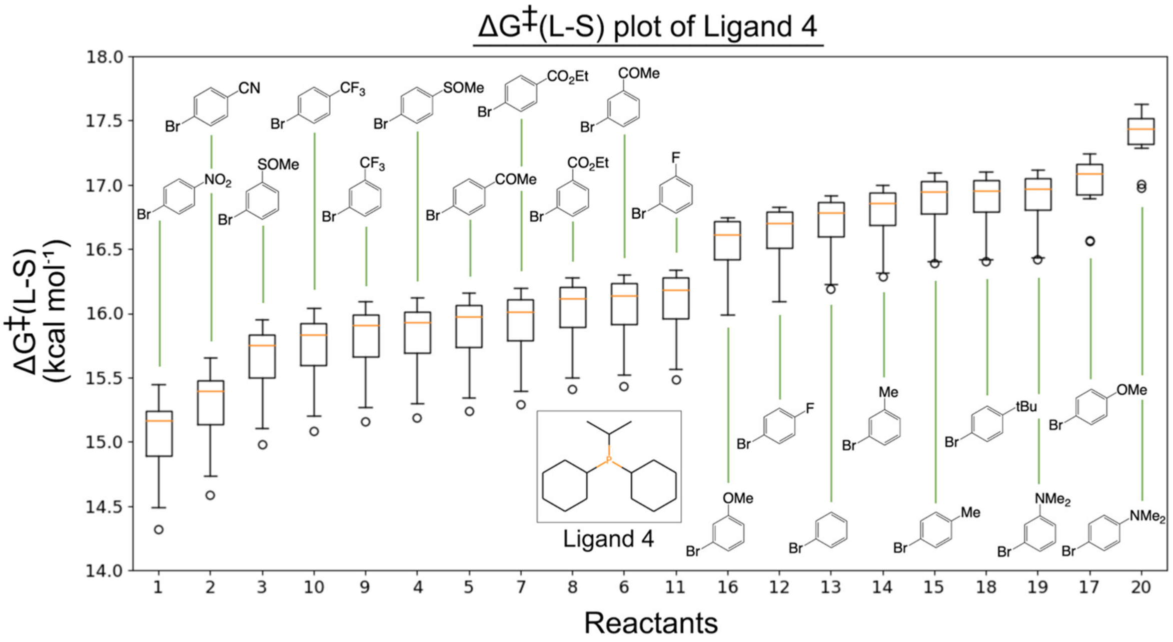

Figure S4. Plot of ΔG‡(L-S) against meta- and para-substituted arylbromide for ligand 4 (dicyclohexyl(propan-2-yl)phosphane).

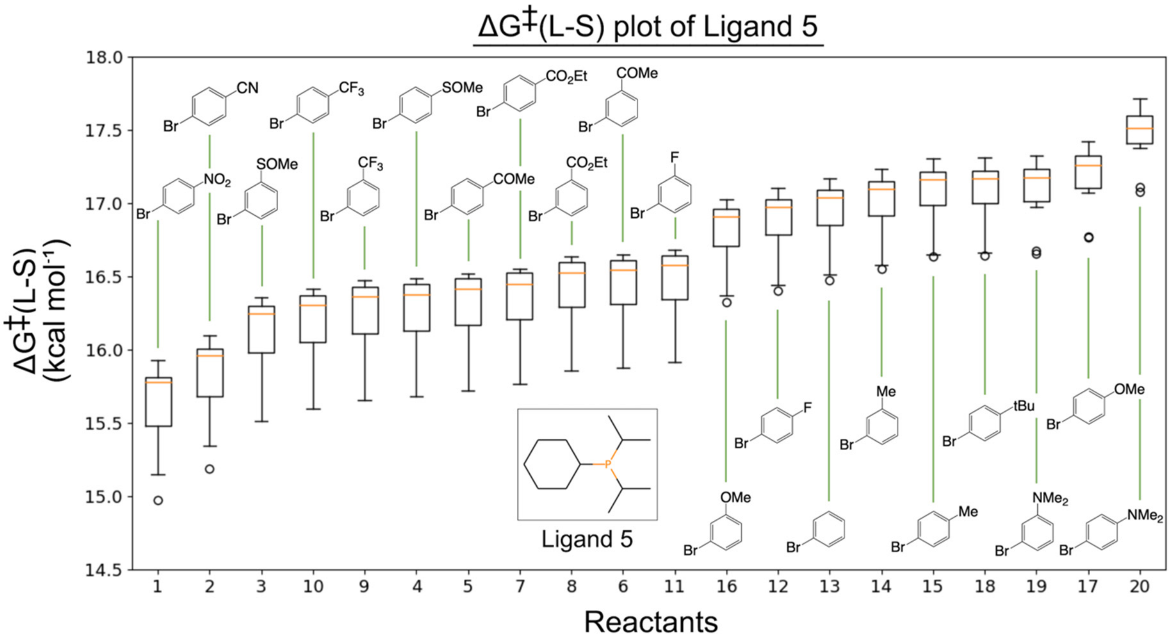

Figure S5. Plot of ΔG‡(L-S) against meta- and para-substituted arylbromide for ligand 5 (cyclohexyl-di(propan-2-yl)phosphane).

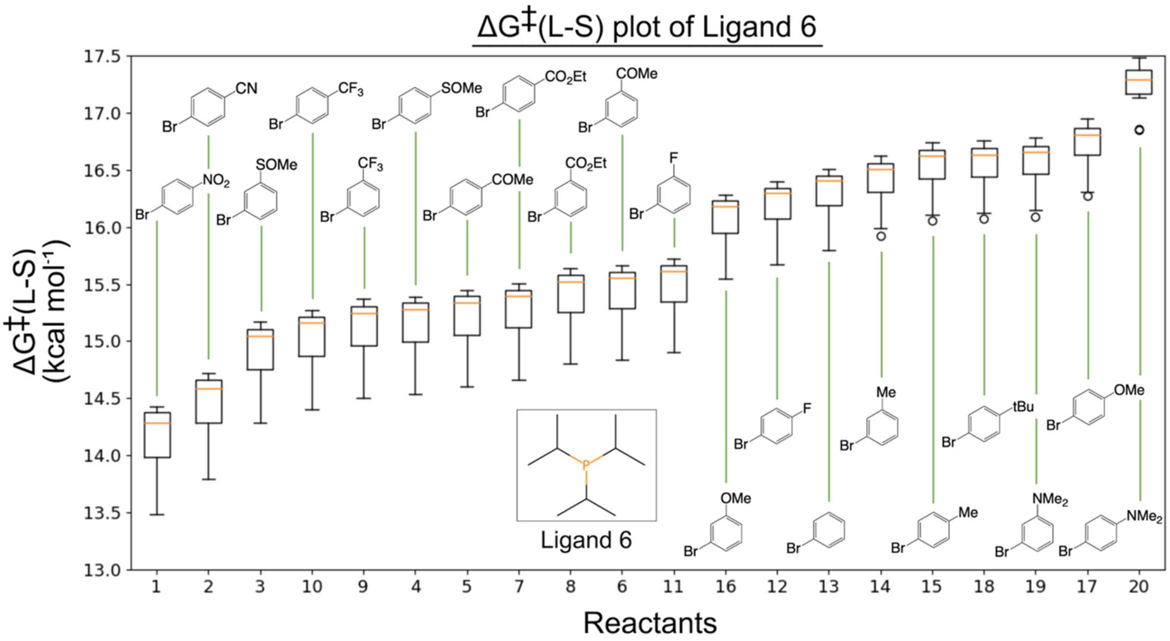

Figure S6. Plot of ΔG‡(L-S) against meta- and para-substituted arylbromide for ligand 6 (tri(propan-2-yl)phosphane).

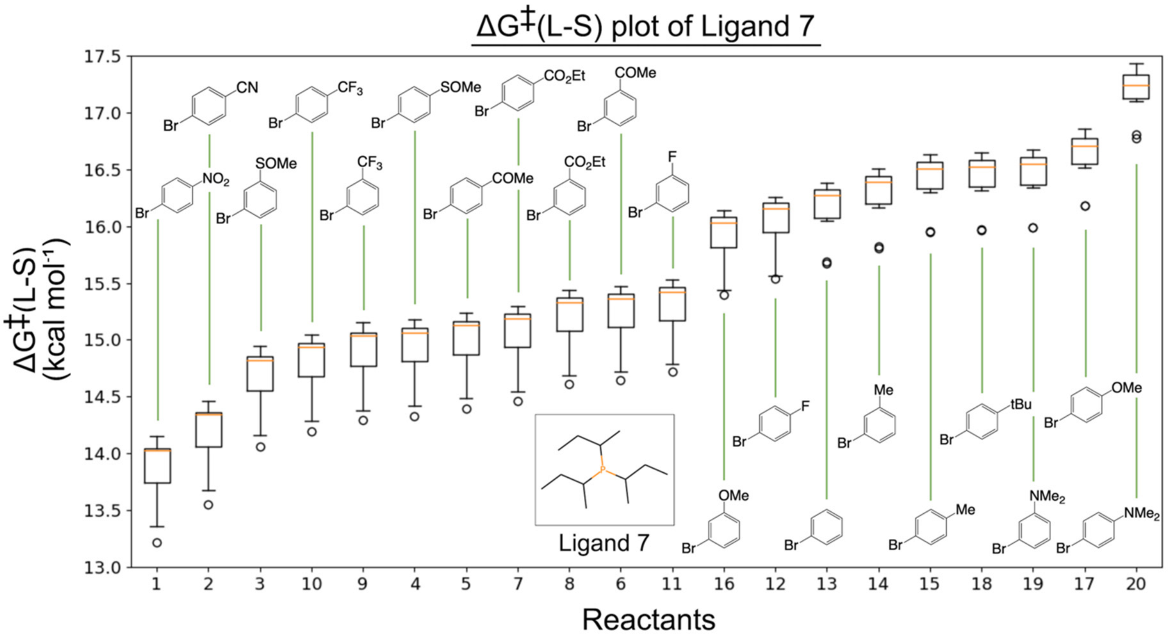

Figure S7. Plot of ΔG‡(L-S) against meta- and para-substituted arylbromide for ligand 7 (tri(butan-2-yl)phosphane).

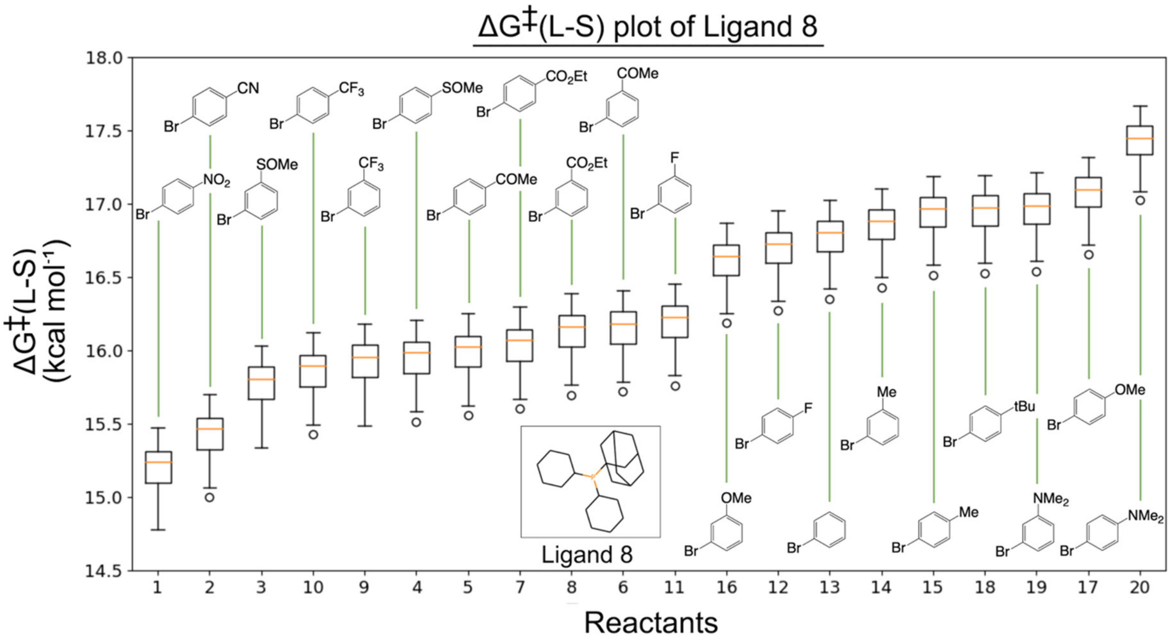

Figure S8. Plot of ΔG‡(L-S) against meta- and para-substituted arylbromide for ligand 8 (1-adamantyl(dicyclohexyl)phosphane).

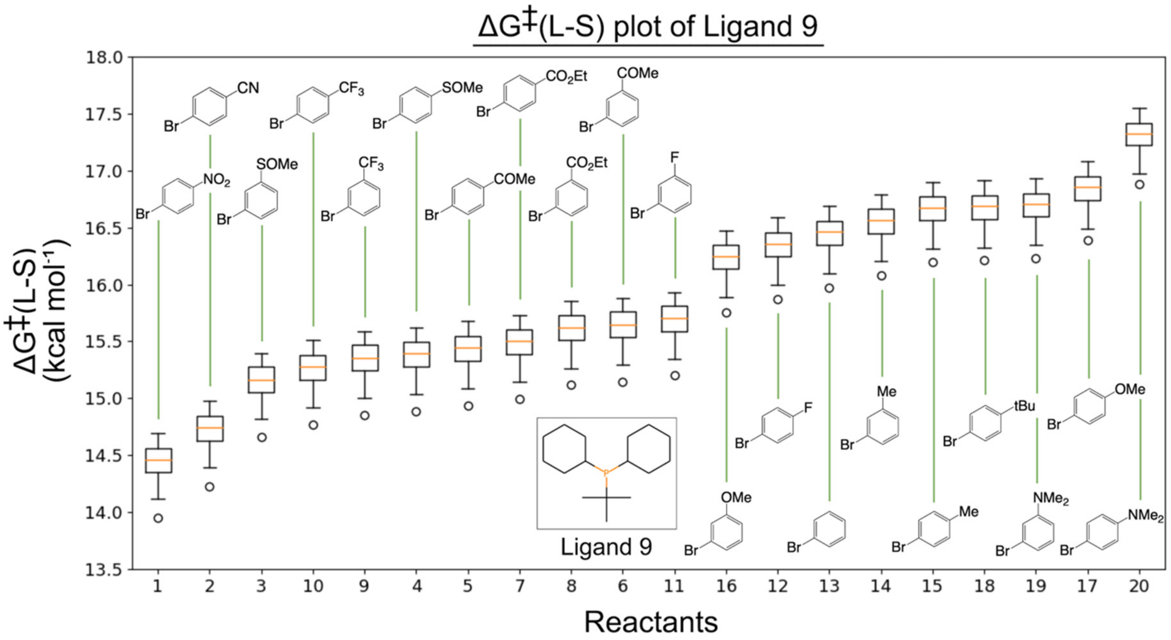

Figure S9. Plot of ΔG‡(L-S) against meta- and para-substituted arylbromide for ligand 9 (tert-butyl(dicyclohexyl)phosphane).

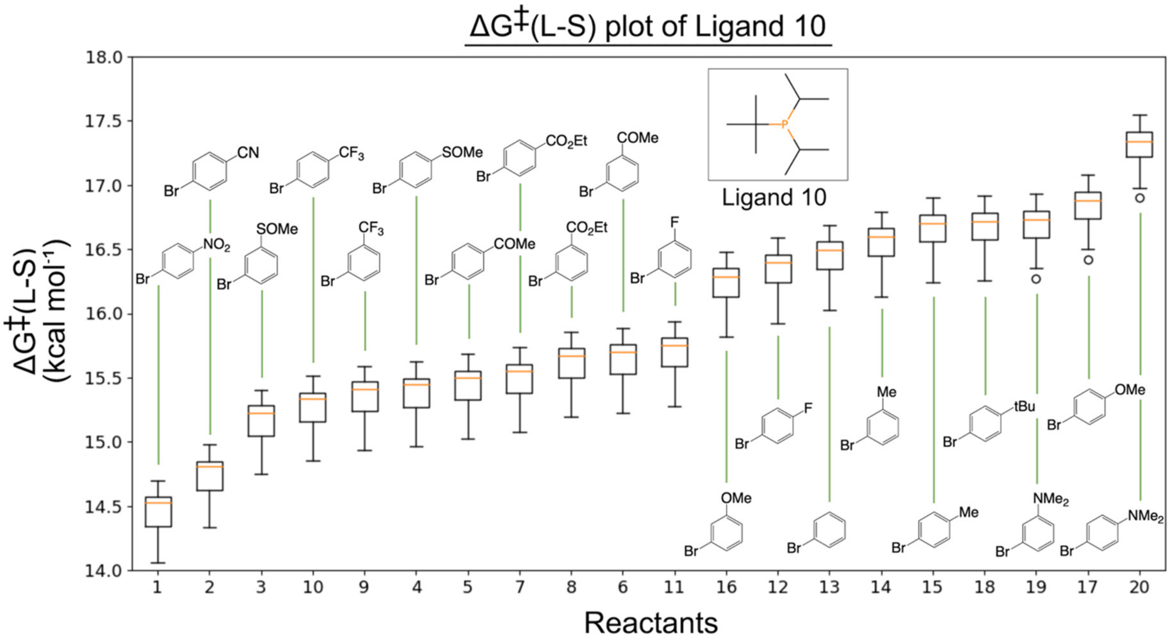

Figure S10. Plot of ΔG‡(L-S) against meta- and para-substituted arylbromide for ligand 10 (tert-butyl-di(propan-2-yl)phosphane).

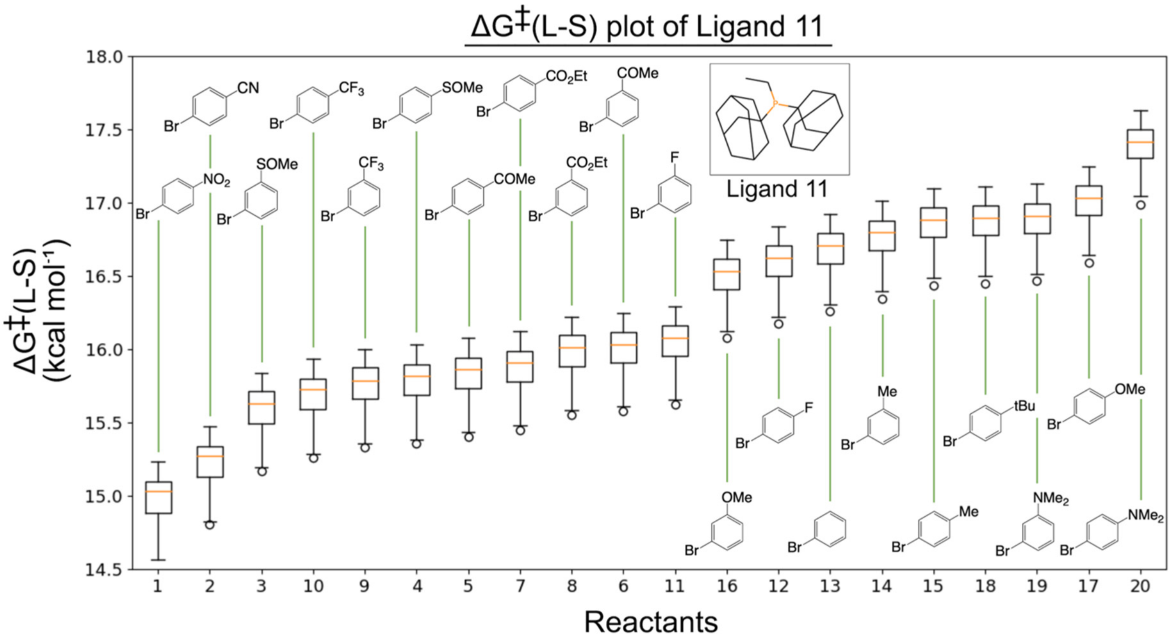

Figure S11. Plot of ΔG‡(L-S) against meta- and para-substituted arylbromide for ligand 11 (bis(1-adamantyl)-ethylphosphane).

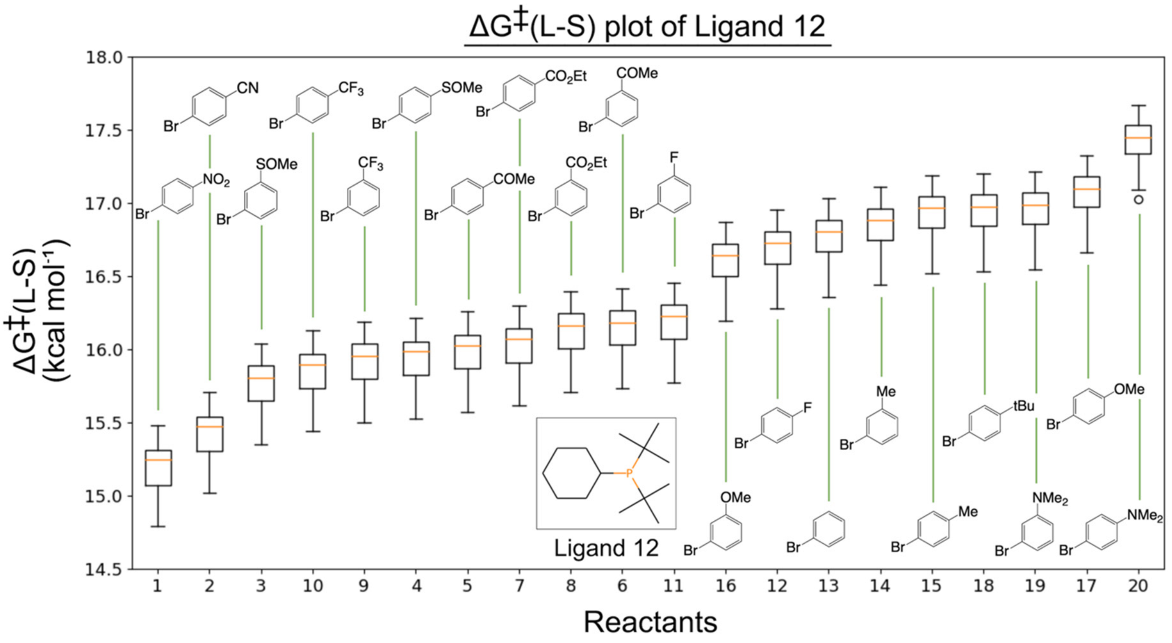

Figure S12. Plot of ΔG‡(L-S) against meta- and para-substituted arylbromide for ligand 12 (ditert-butyl(cyclohexyl)phosphane).

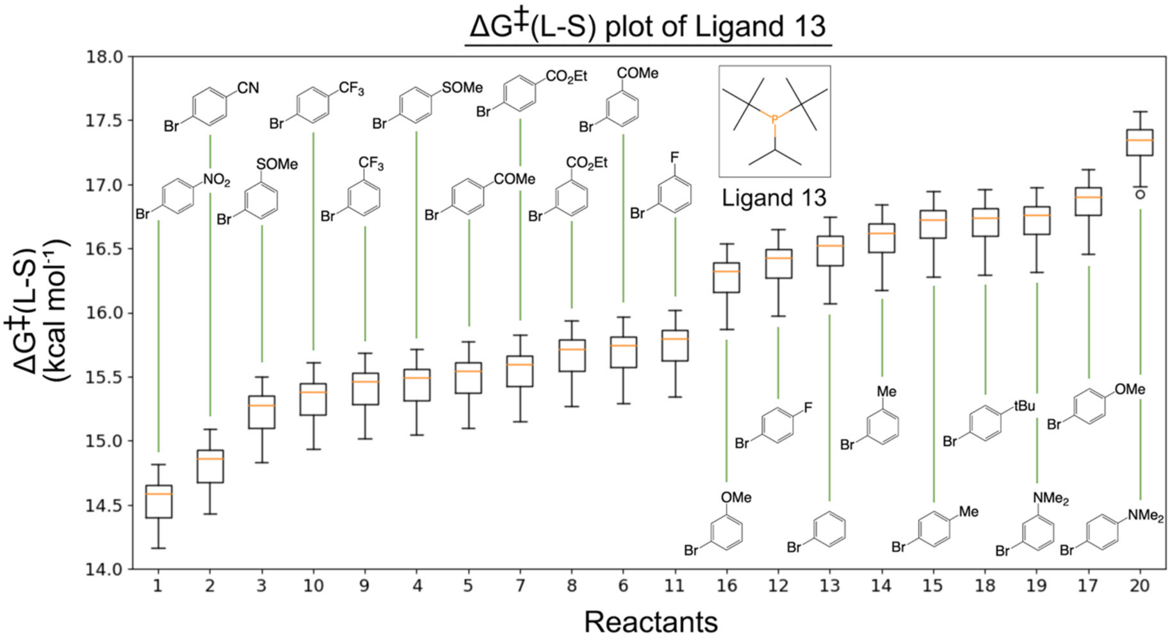

Figure S13. Plot of ΔG‡(L-S) against meta- and para-substituted arylbromide for ligand 13 (ditert-butyl(propan-2-yl)phosphane).

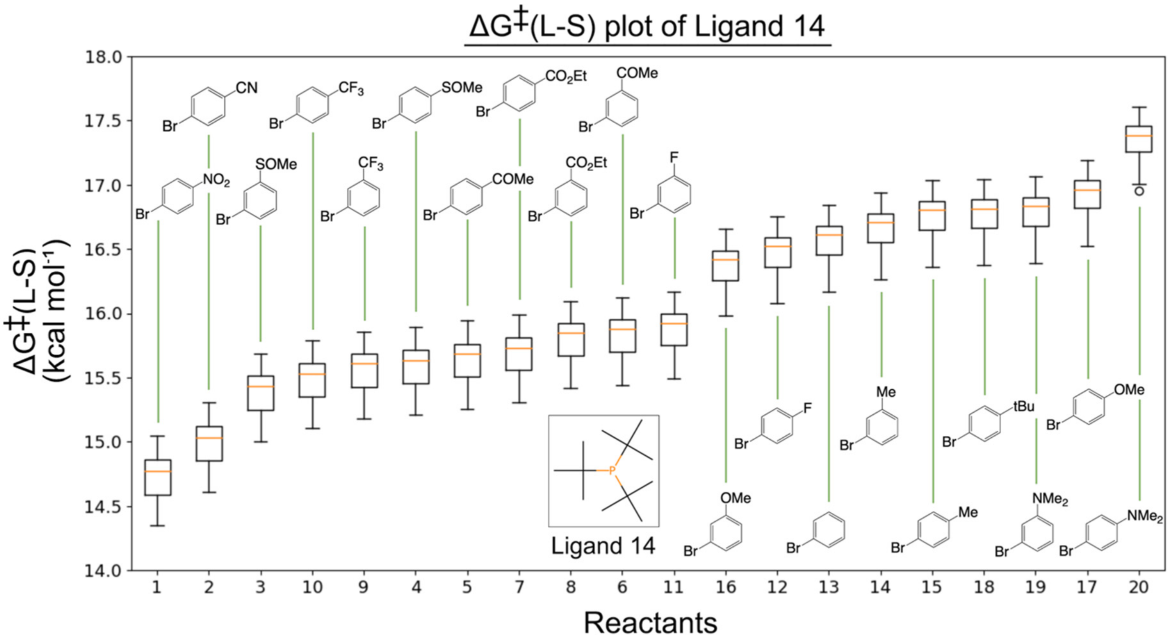

Figure S14. Plot of ΔG‡(L-S) against meta- and para-substituted arylbromide for ligand 14 (tritert-butylphosphane).

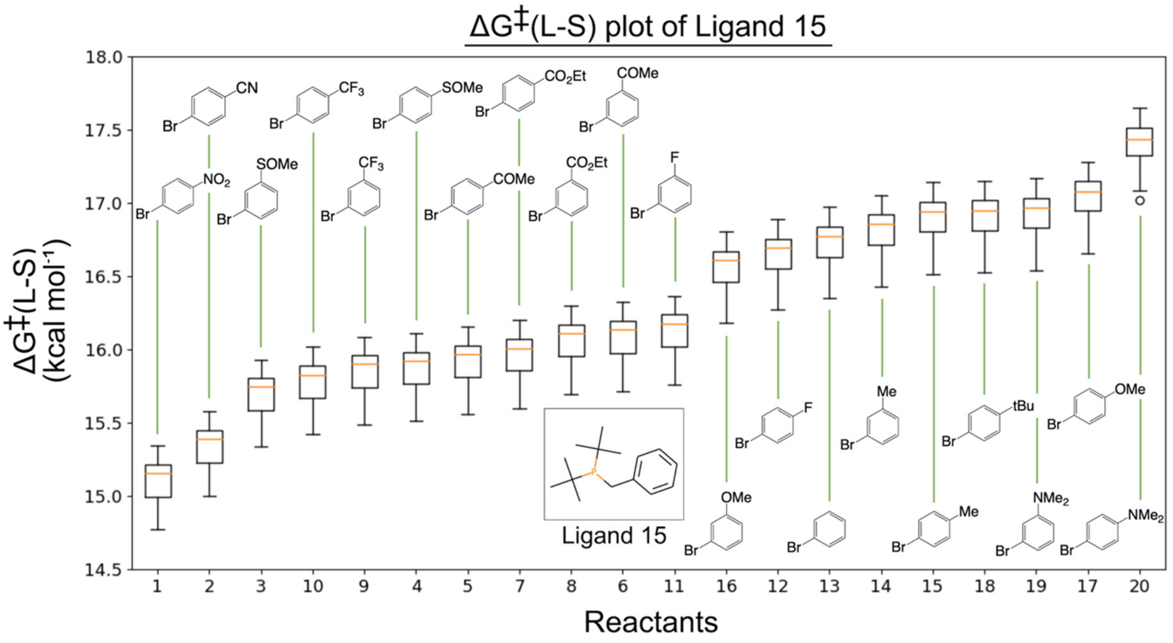

Figure S15. Plot of ΔG‡(L-S) against meta- and para-substituted arylbromide for ligand 15 (benzyl(ditert-butyl)phosphane).

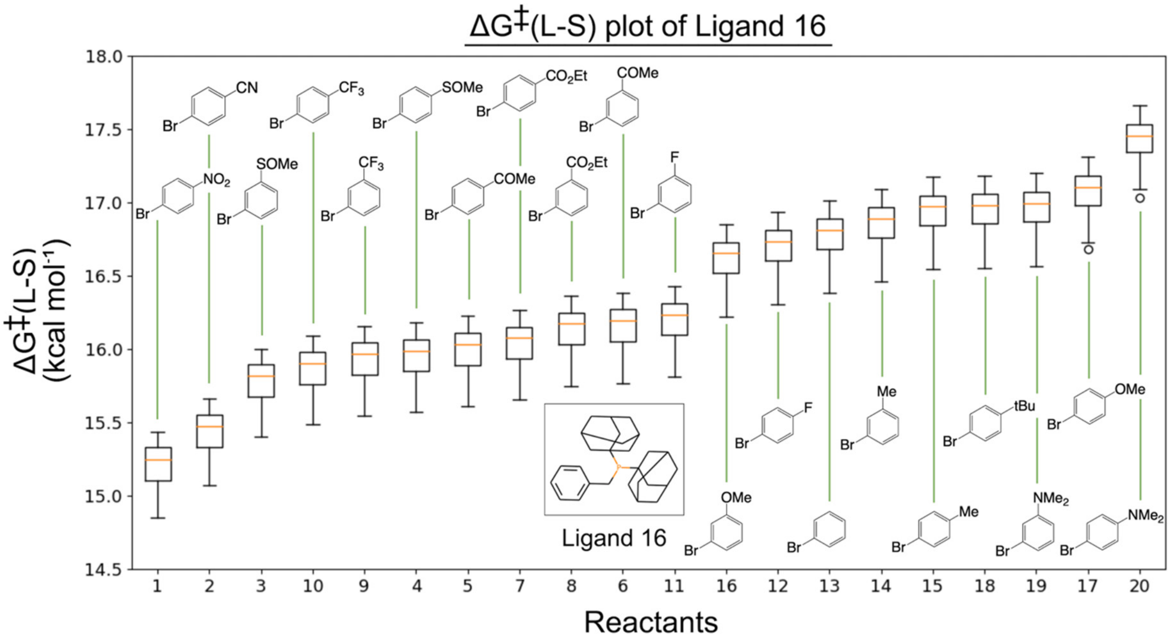

Figure S16. Plot of ΔG‡(L-S) against meta- and para-substituted arylbromide for ligand 16 (bis(1-adamantyl)-benzylphosphane).

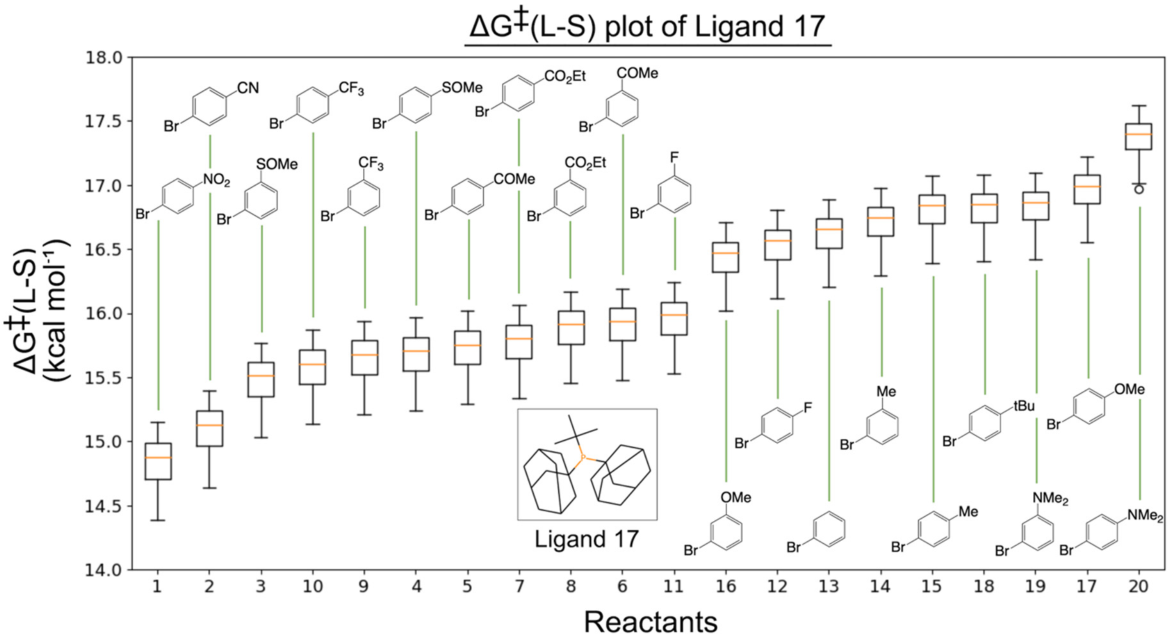

Figure S17. Plot of ΔG‡(L-S) against meta- and para-substituted arylbromide for ligand 17 (bis(1-adamantyl)-tert-butylphosphane).

2. Plots of ΔG‡(L-S) against ligand for Arylbromides 1-20

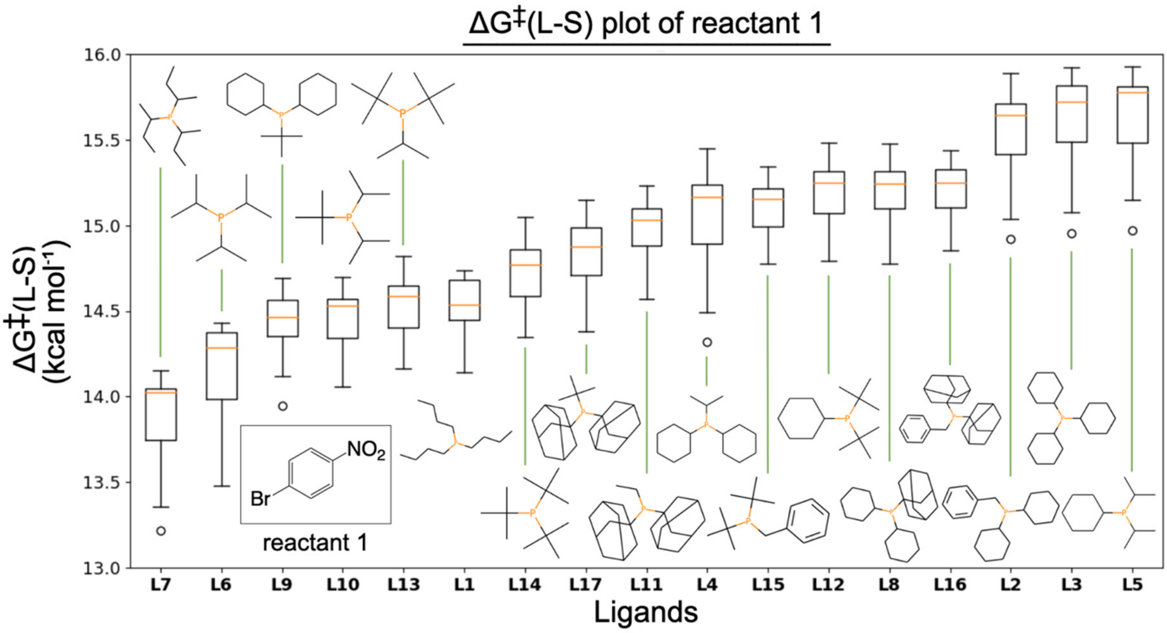

Figure S18. Plot of ΔG‡(L-S) against ligand for 1-bromo-4-nitrobenzene.

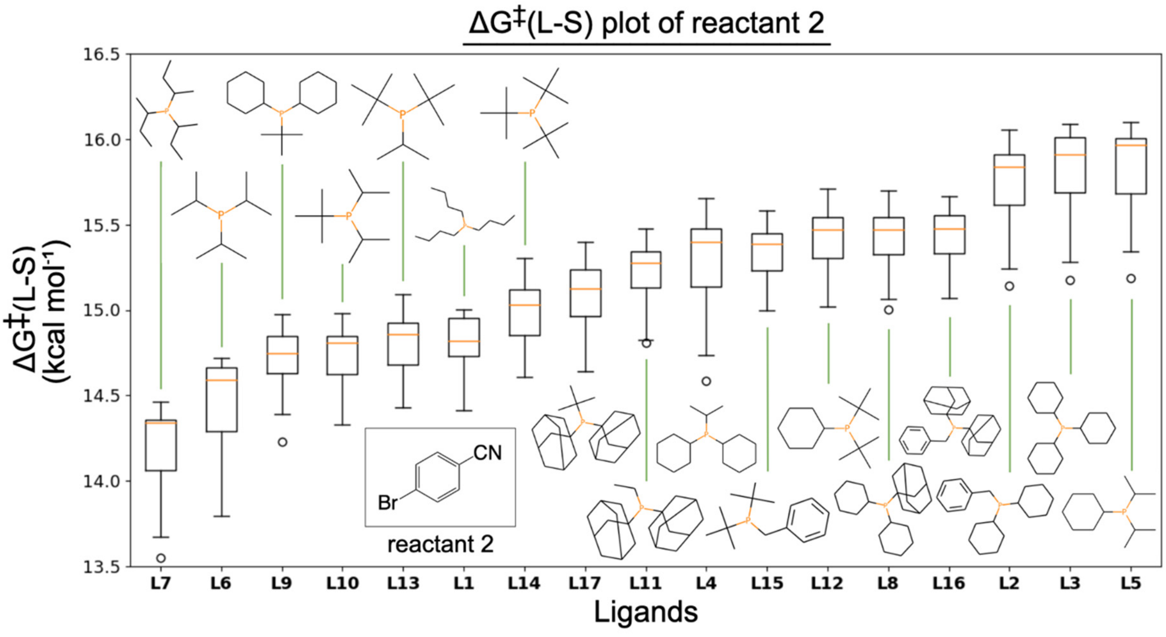

Figure S19. Plot of ΔG‡(L-S) against ligand for 4-bromobenzonitrile.

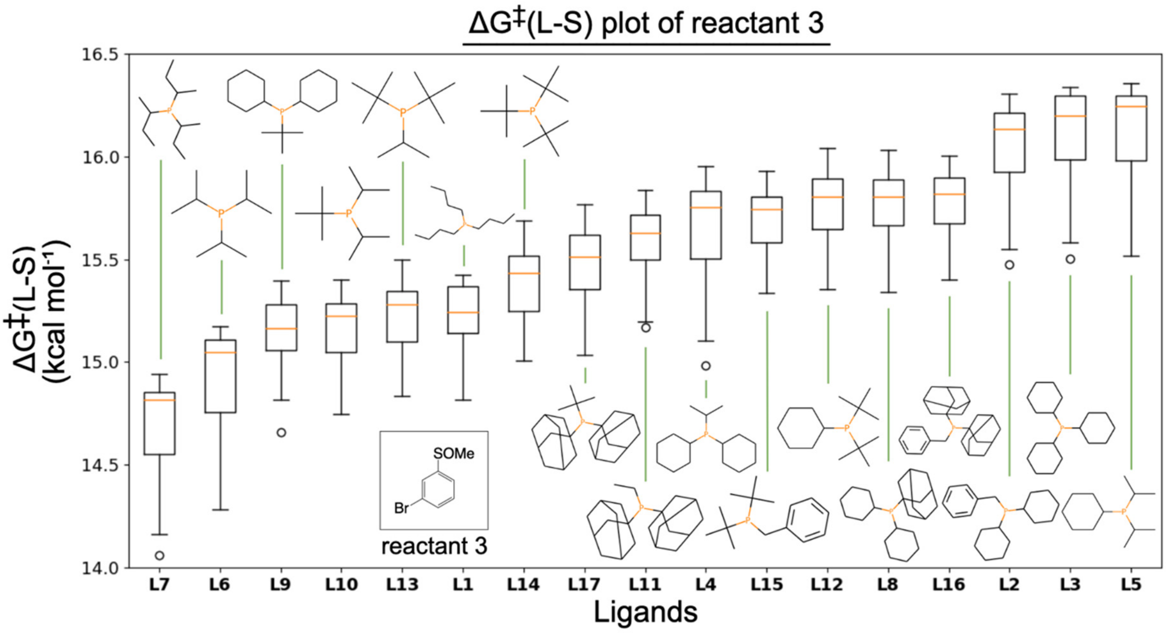

Figure S20. Plot of ΔG‡(L-S) against ligand for 1-bromo-3-methylsulfinylbenzene.

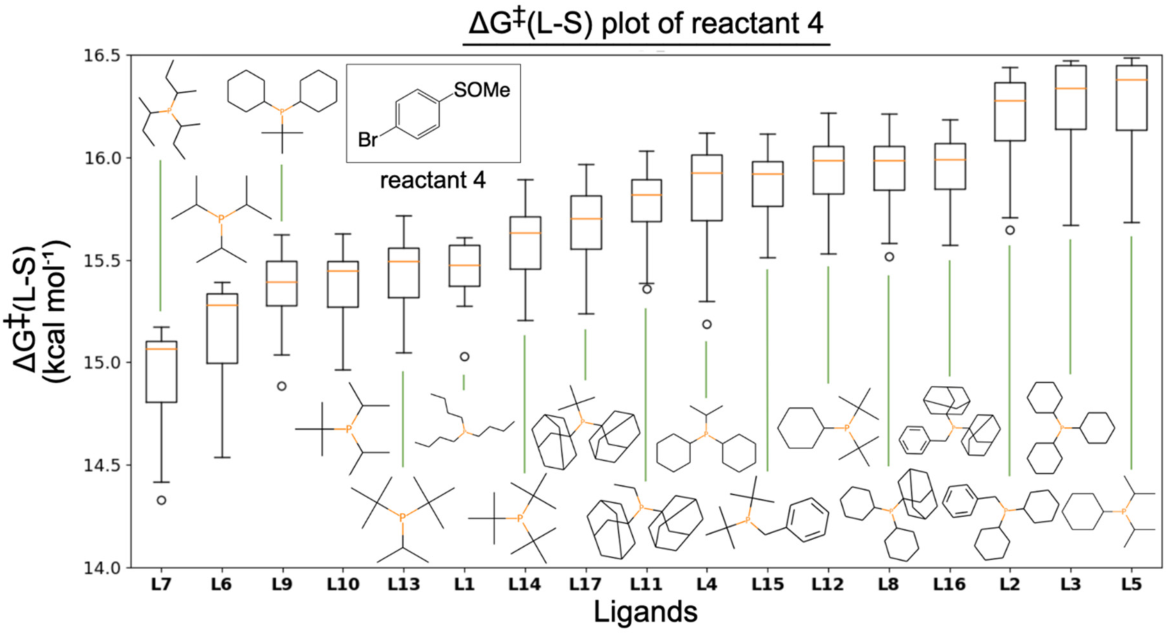

Figure S21. Plot of ΔG‡(L-S) against ligand for 1-bromo-4-methylsulfinylbenzene.

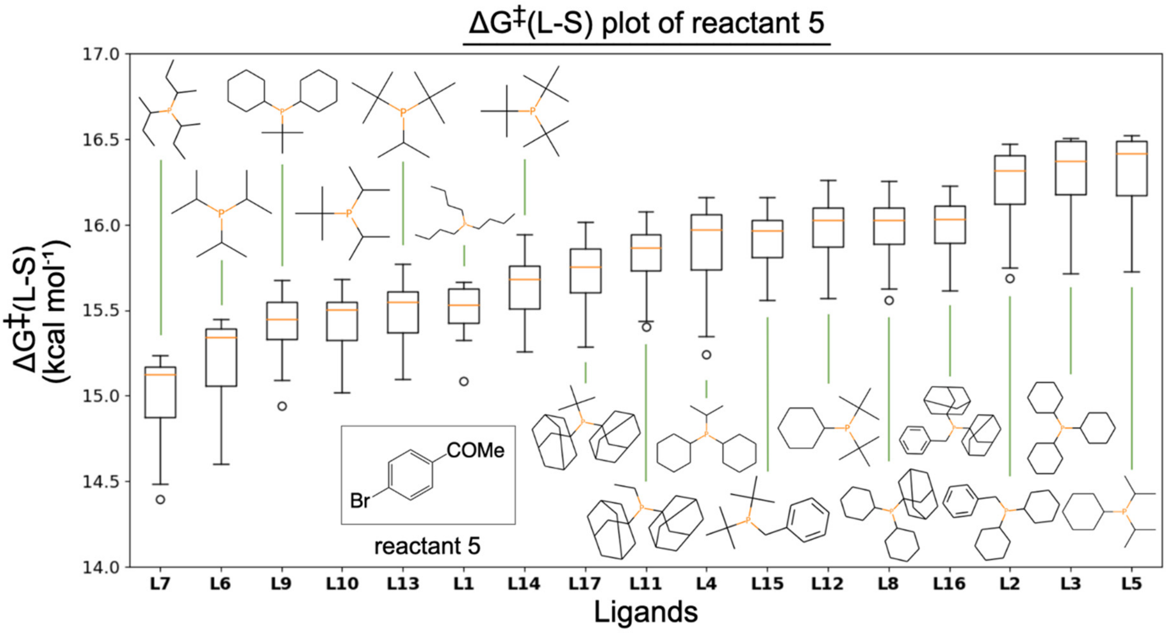

Figure S22. Plot of ΔG‡(L-S) against ligand for 1-(4-bromophenyl)ethanone.

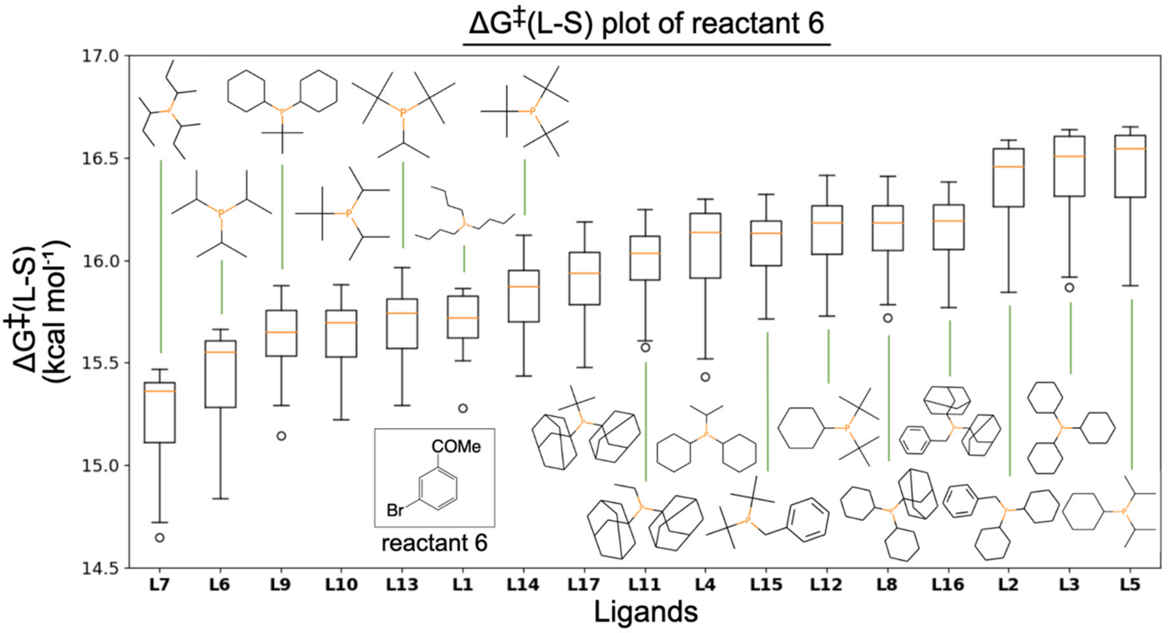

Figure S23. Plot of ΔG‡(L-S) against ligand for 1-(3-bromophenyl)ethanone.

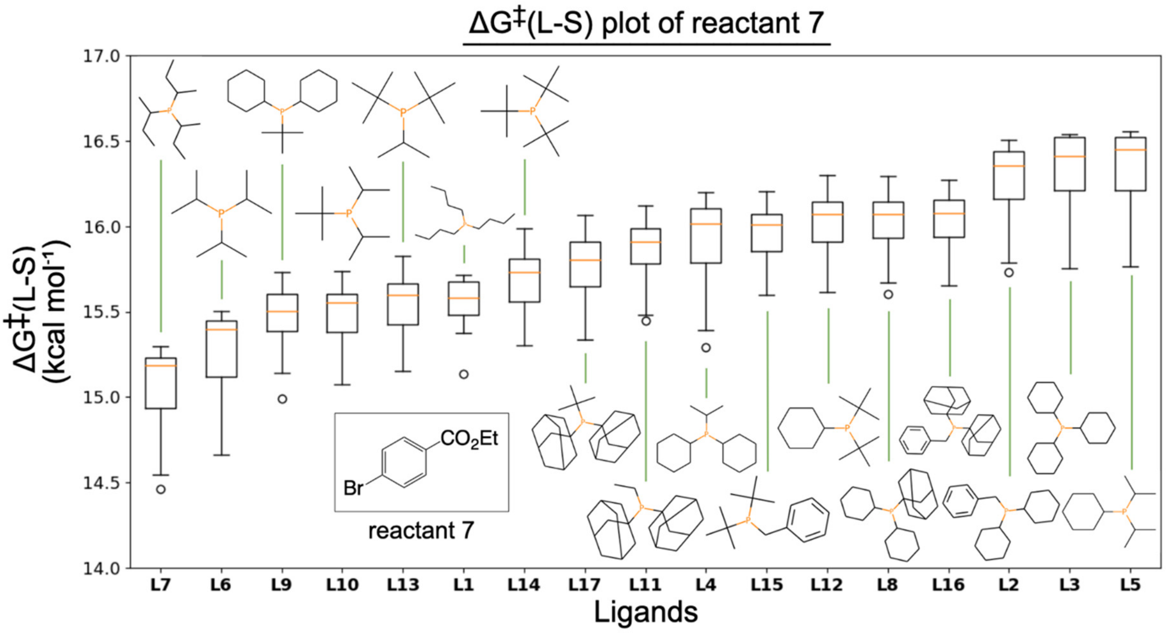

Figure S24. Plot of ΔG‡(L-S) against ligand for ethyl 4-bromobenzoate.

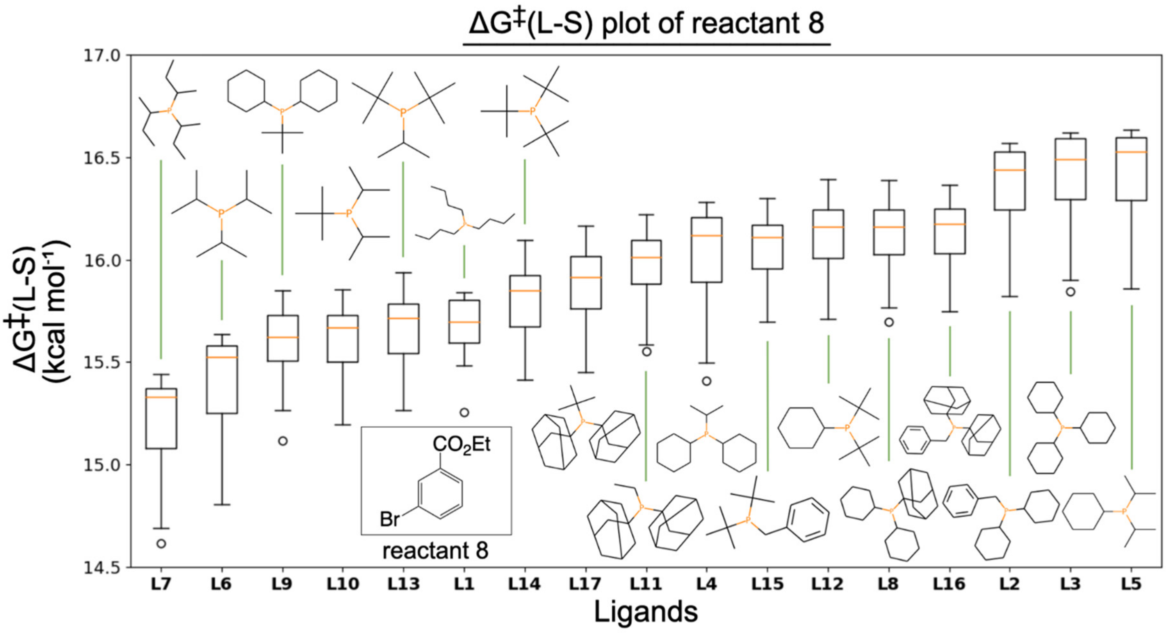

Figure S25. Plot of ΔG‡(L-S) against ligand for ethyl 3-bromobenzoate.

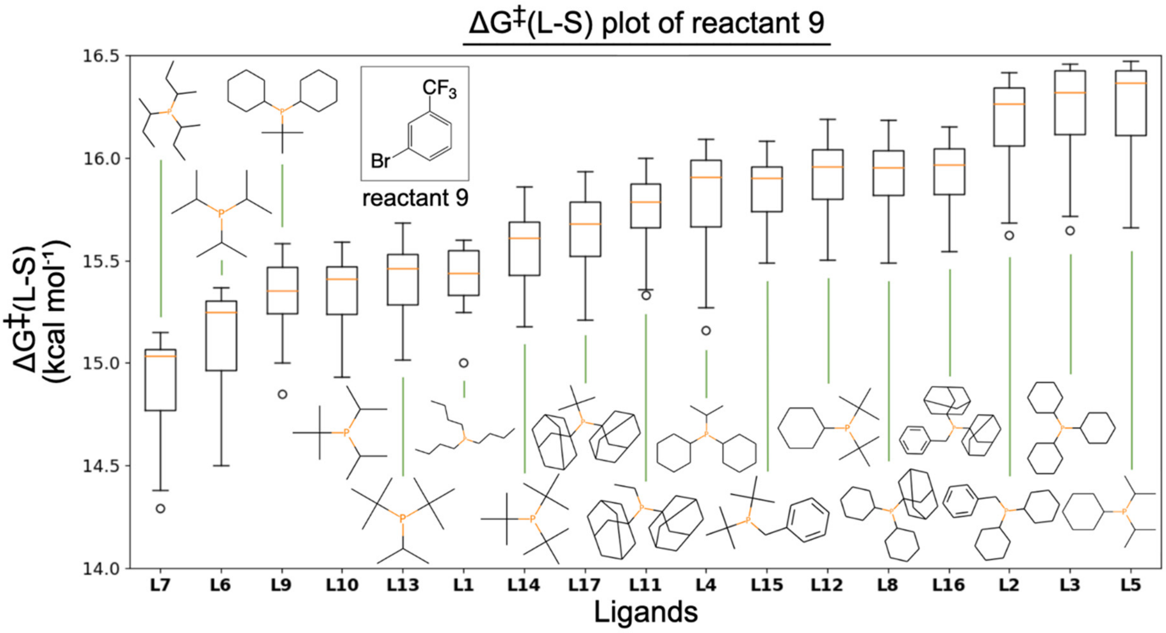

Figure S26. Plot of ΔG‡(L-S) against ligand for 1-bromo-3-(trifluoromethyl)benzene.

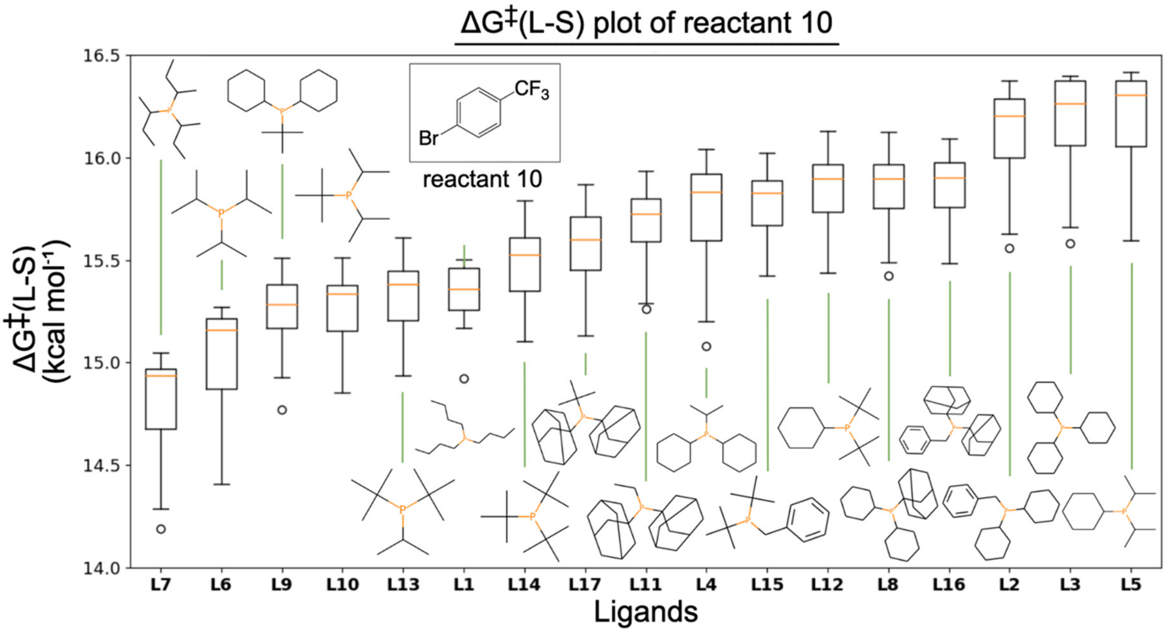

Figure S27. Plot of ΔG‡(L-S) against ligand for 1-bromo-4-(trifluoromethyl)benzene.

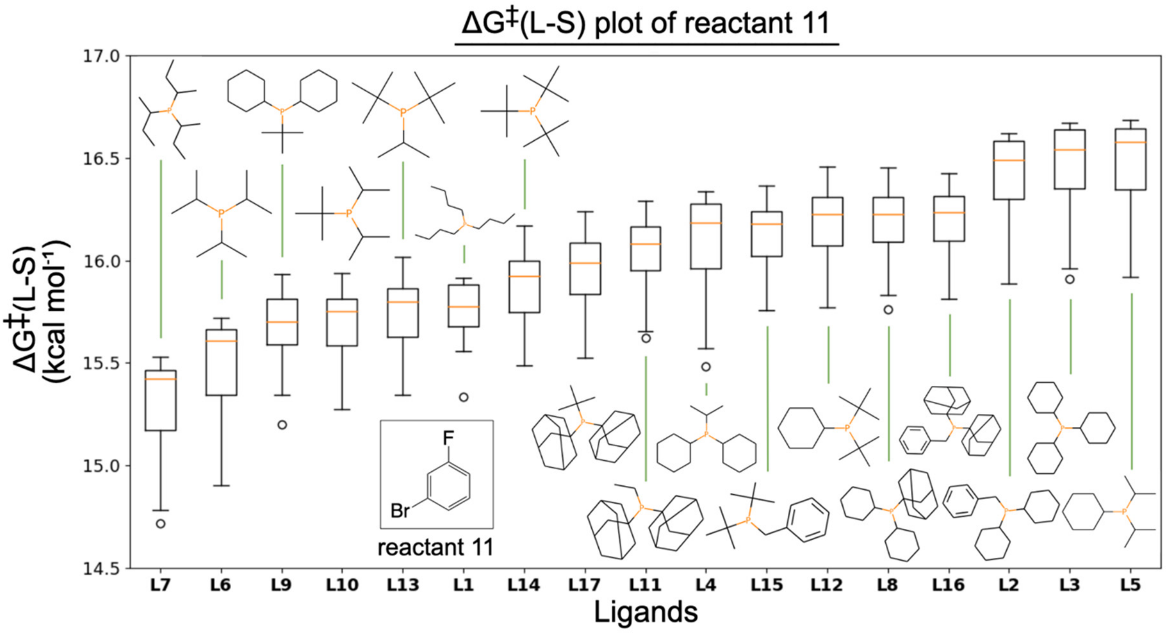

Figure S28. Plot of ΔG‡(L-S) against ligand for1-bromo-3-fluorobenzene.

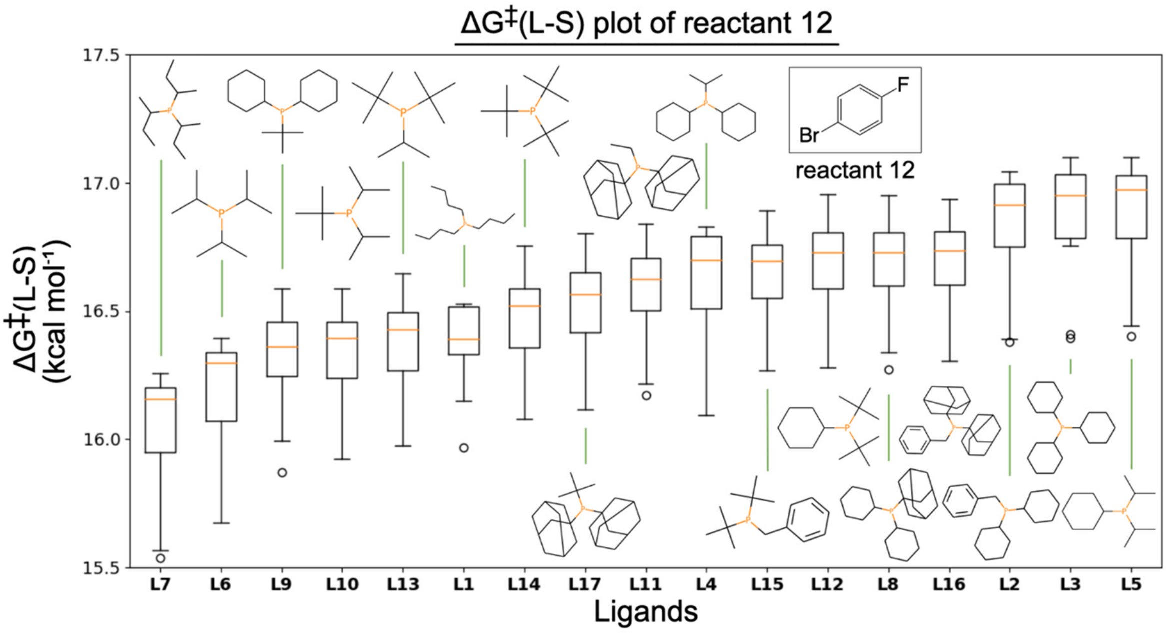

Figure S29. Plot of ΔG‡(L-S) against ligand for 1-bromo-4-fluorobenzene.

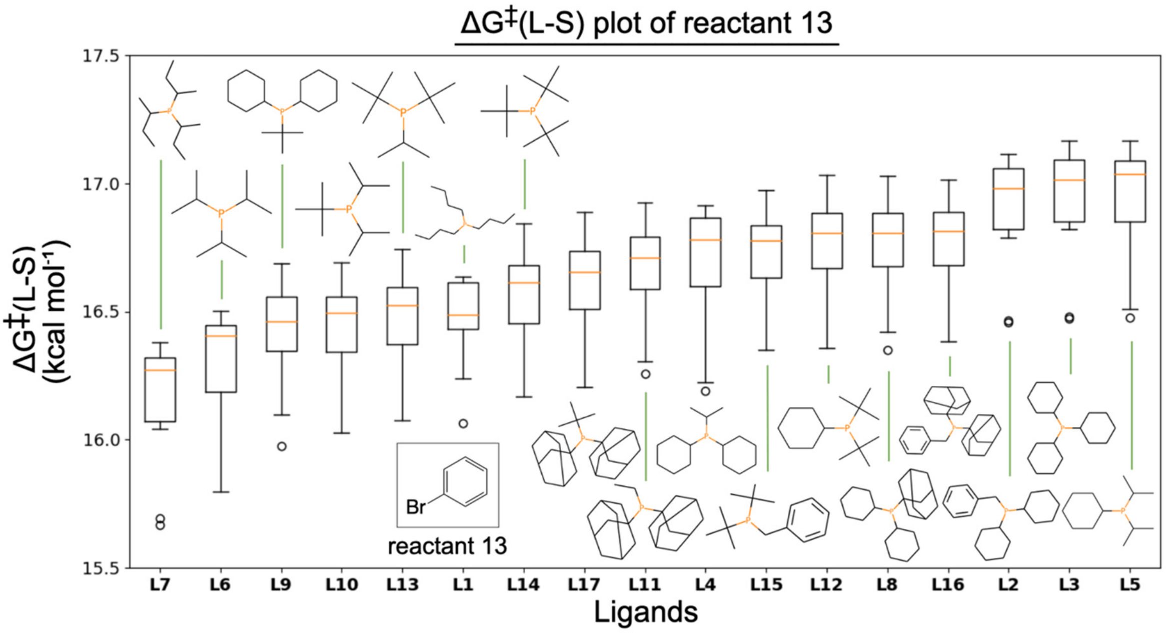

Figure S30. Plot of ΔG‡(L-S) against ligand for bromobenzene.

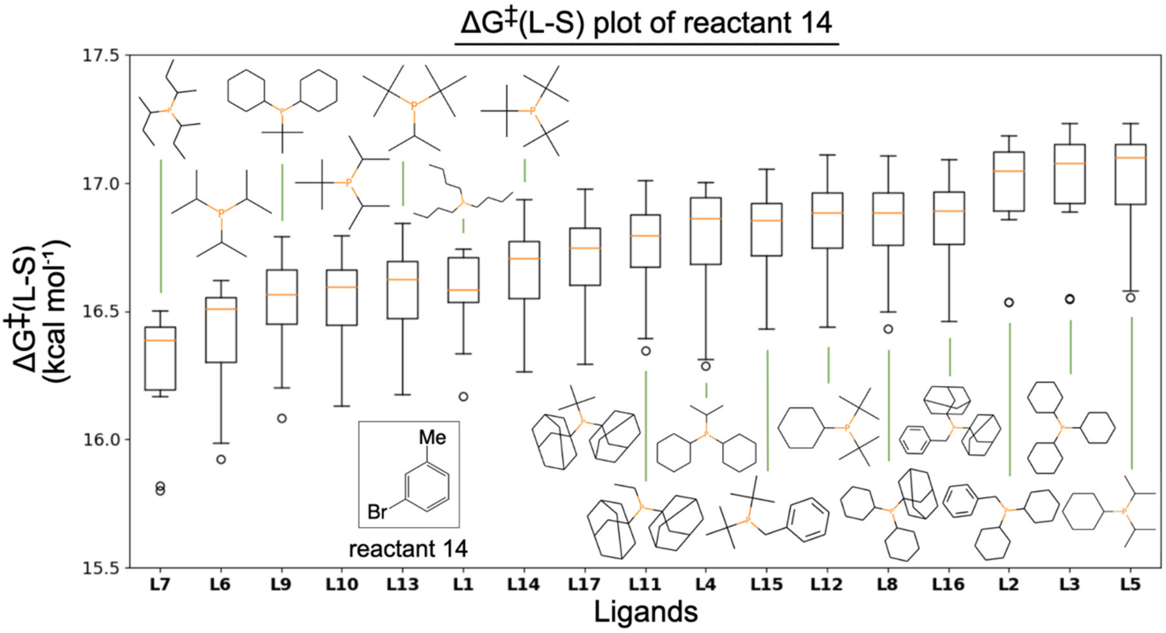

Figure S31. Plot of ΔG‡(L-S) against ligand for 1-bromo-3-methylbenzene.

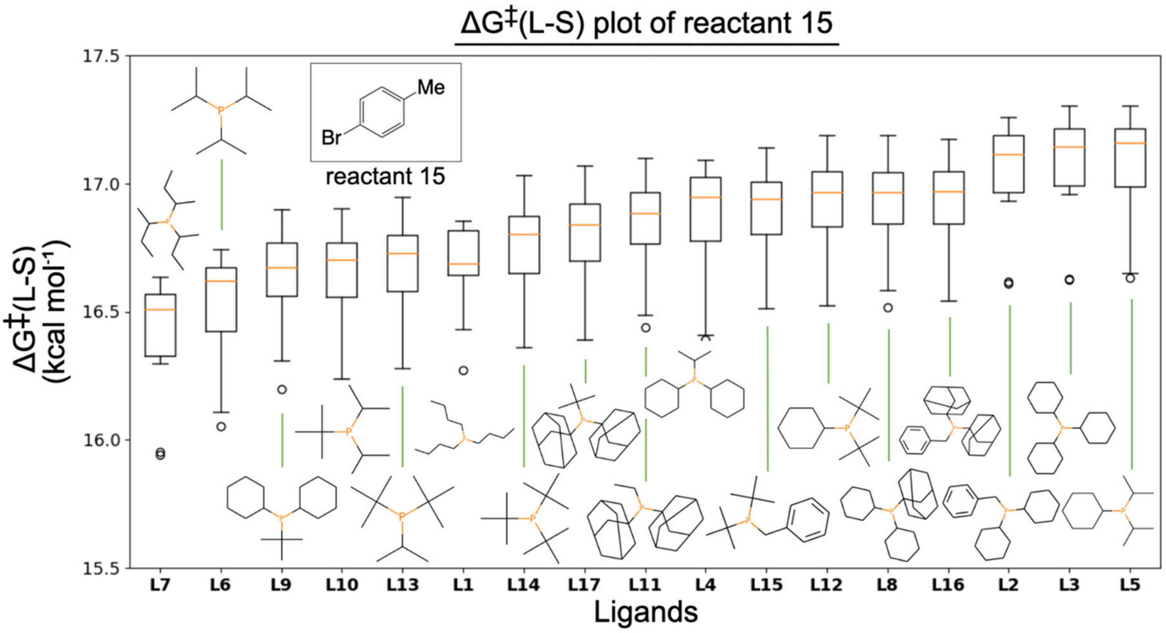

Figure S32. Plot of ΔG‡(L-S) against ligand for 1-bromo-4-methylbenzene.

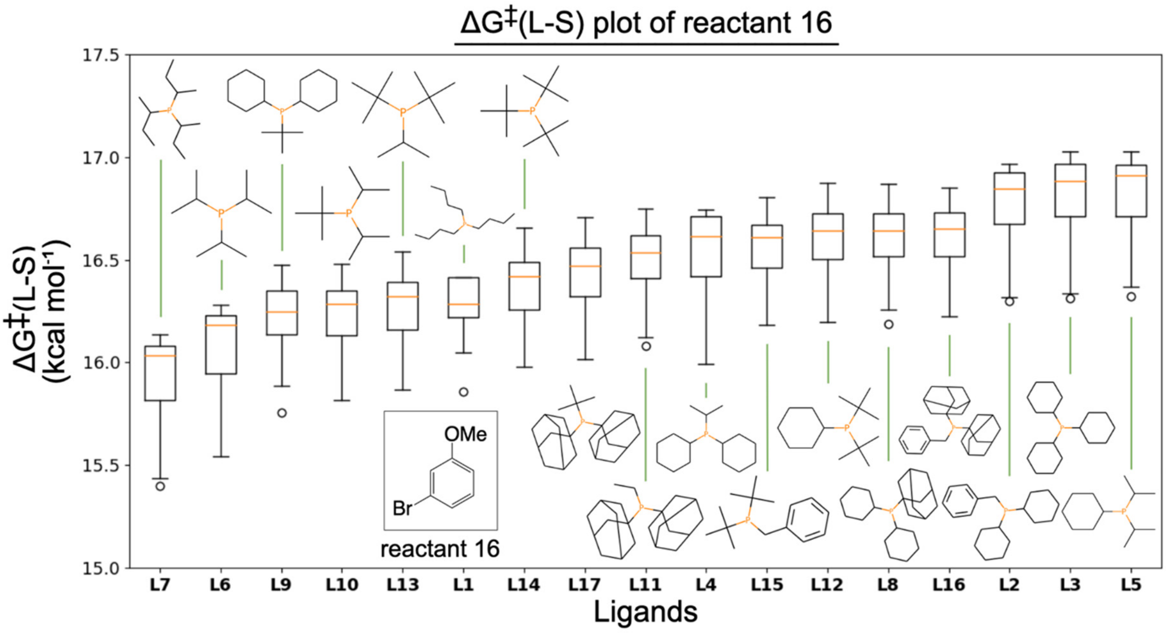

Figure S33. Plot of ΔG‡(L-S) against ligand for 1-bromo-3-methoxybenzene.

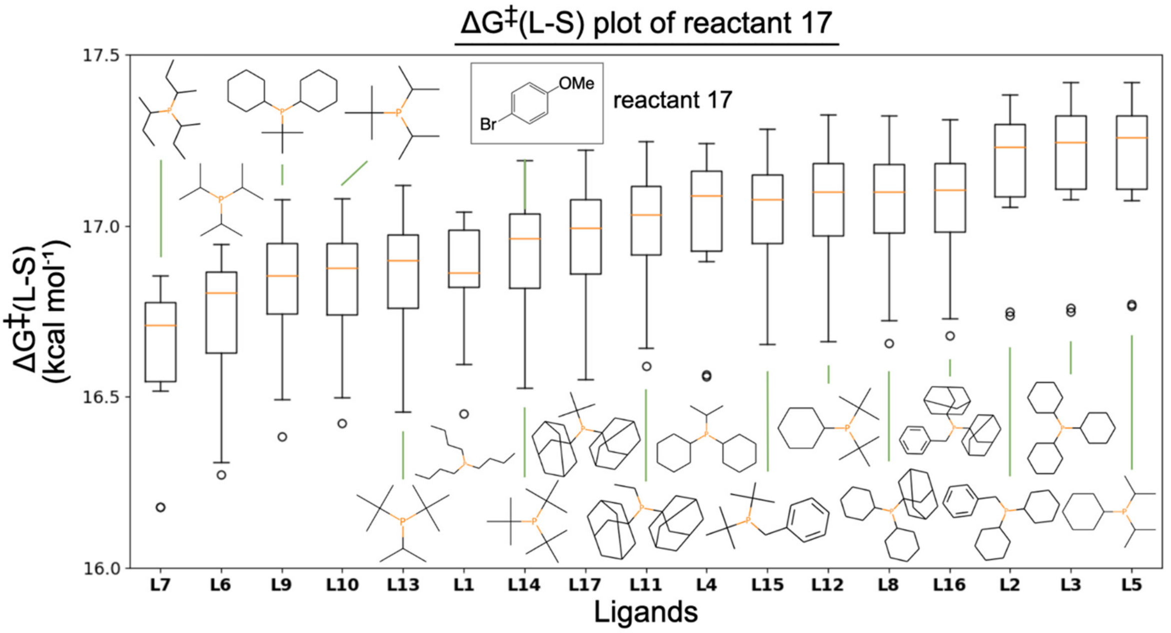

Figure S34. Plot of ΔG‡(L-S) against ligand for 1-bromo-4-methoxybenzene.

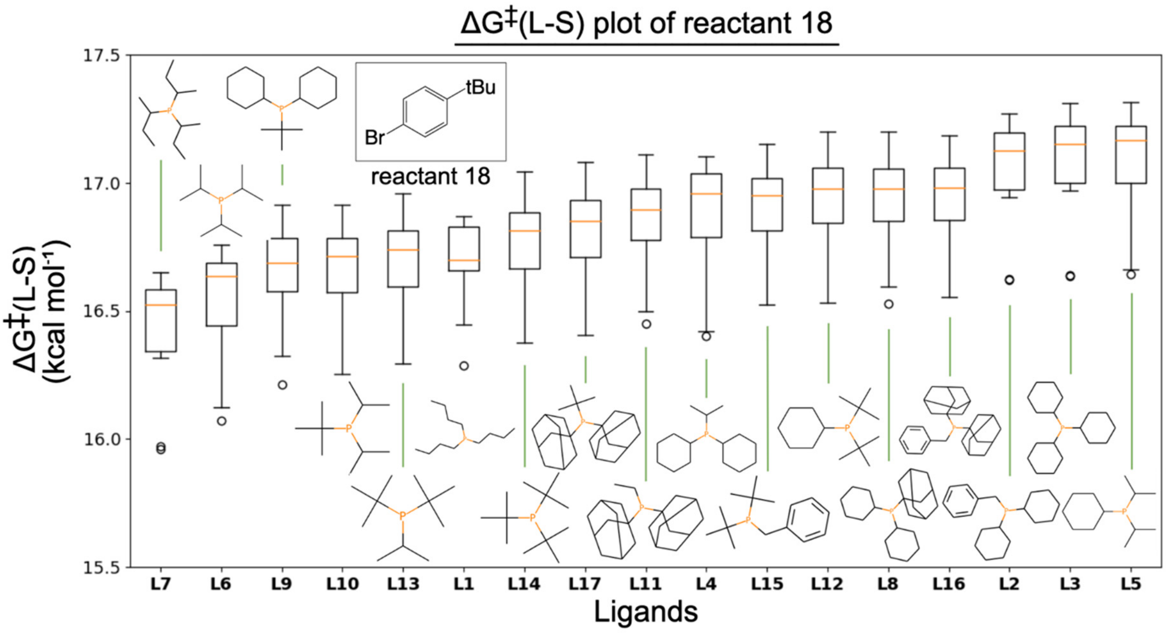

Figure S35. Plot of ΔG‡(L-S) against ligand for 1-bromo-4-tert-butylbenzene.

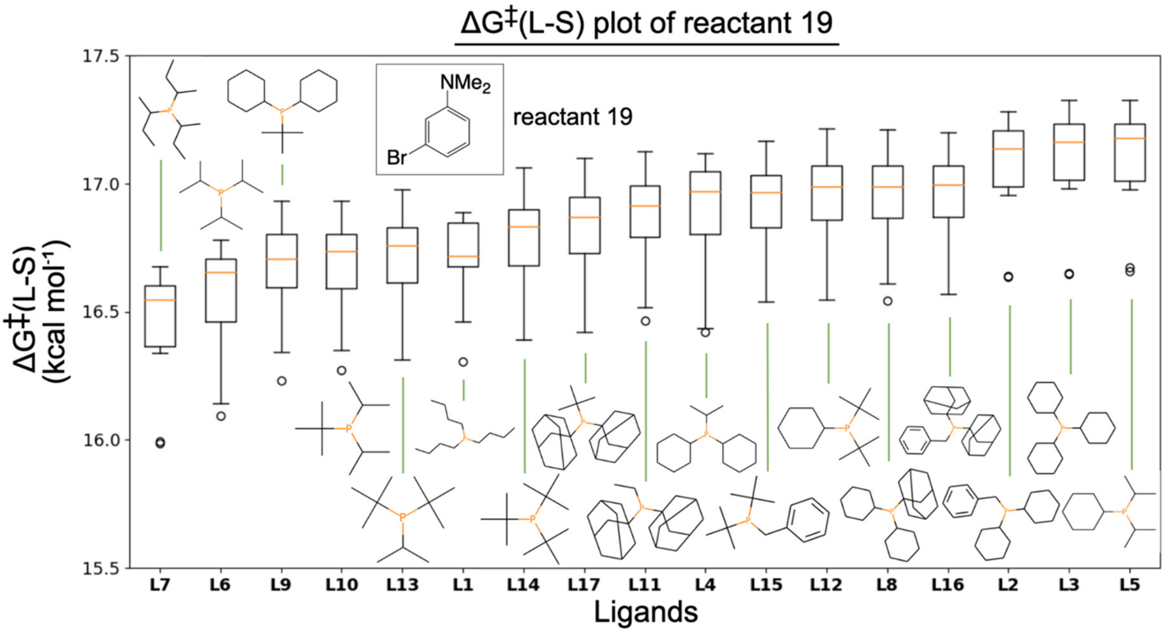

Figure S36. Plot of ΔG‡(L-S) against ligand for 3-bromo-N,N-dimethylaniline.

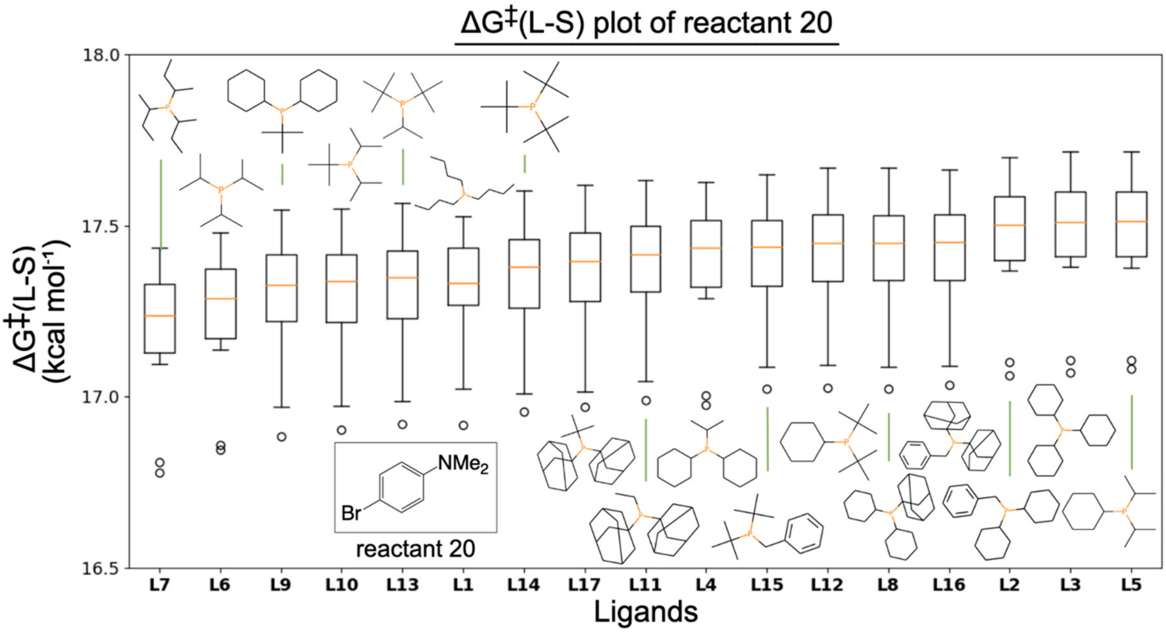

Figure S37. Plot of ΔG‡(L-S) against ligand for 4-bromo-N,N-dimethylaniline.

3. Parity plots for cross validation sets 1-4

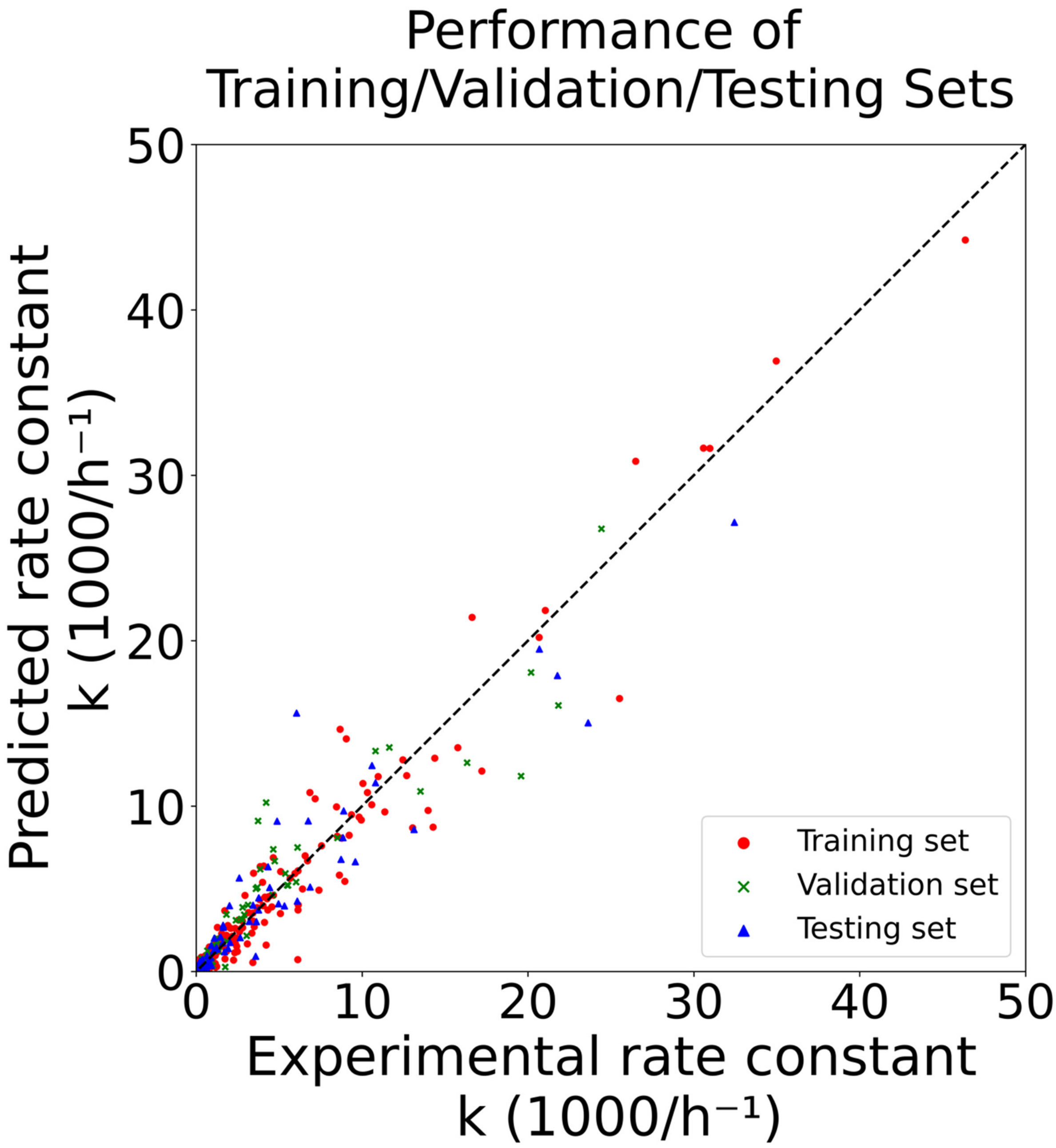

Figure S38. Parity plot of predicted against experimental k for cross validation set 1

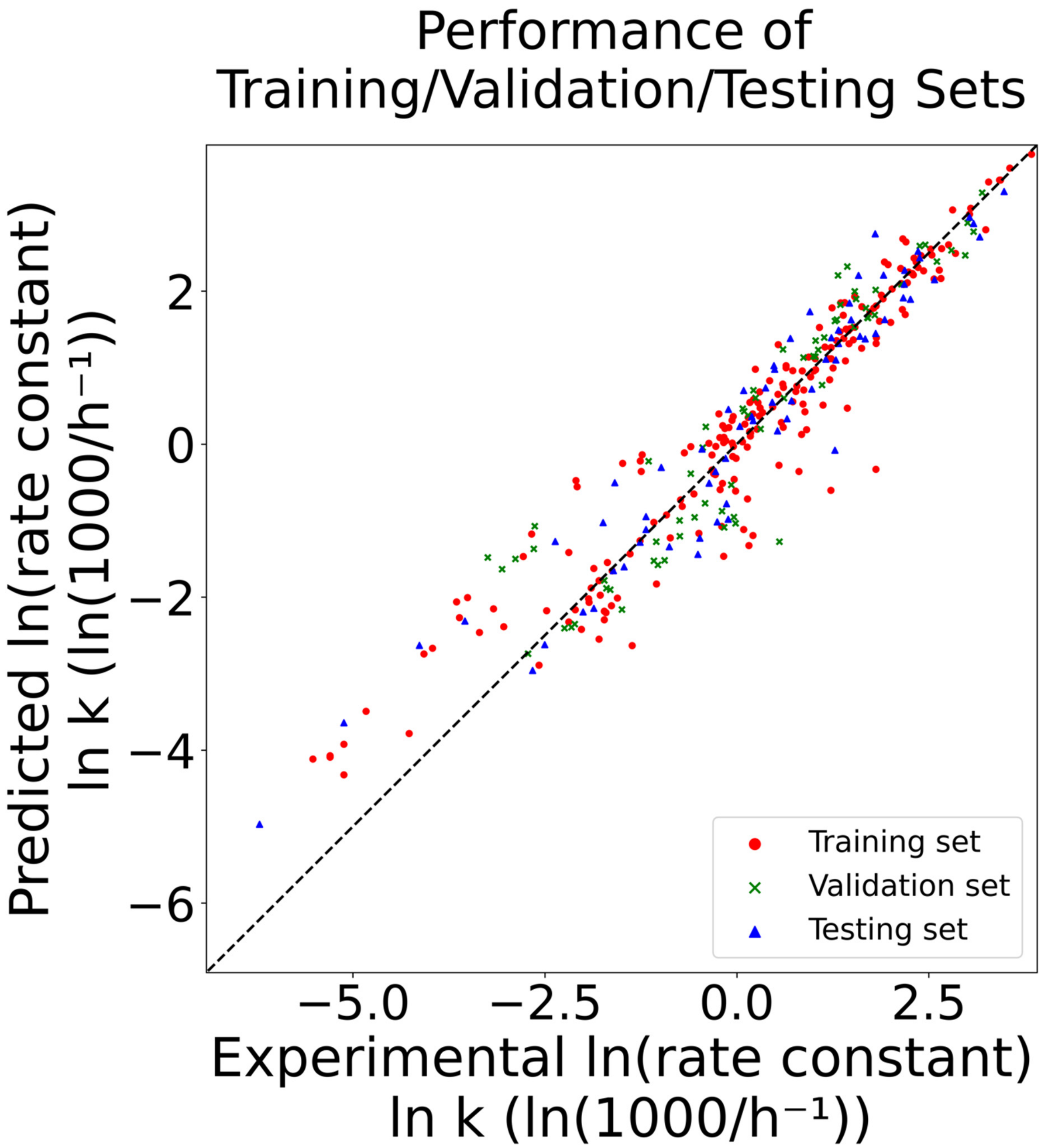

Figure S39. Parity plot of predicted against experimental ln(k) for cross validation set 1

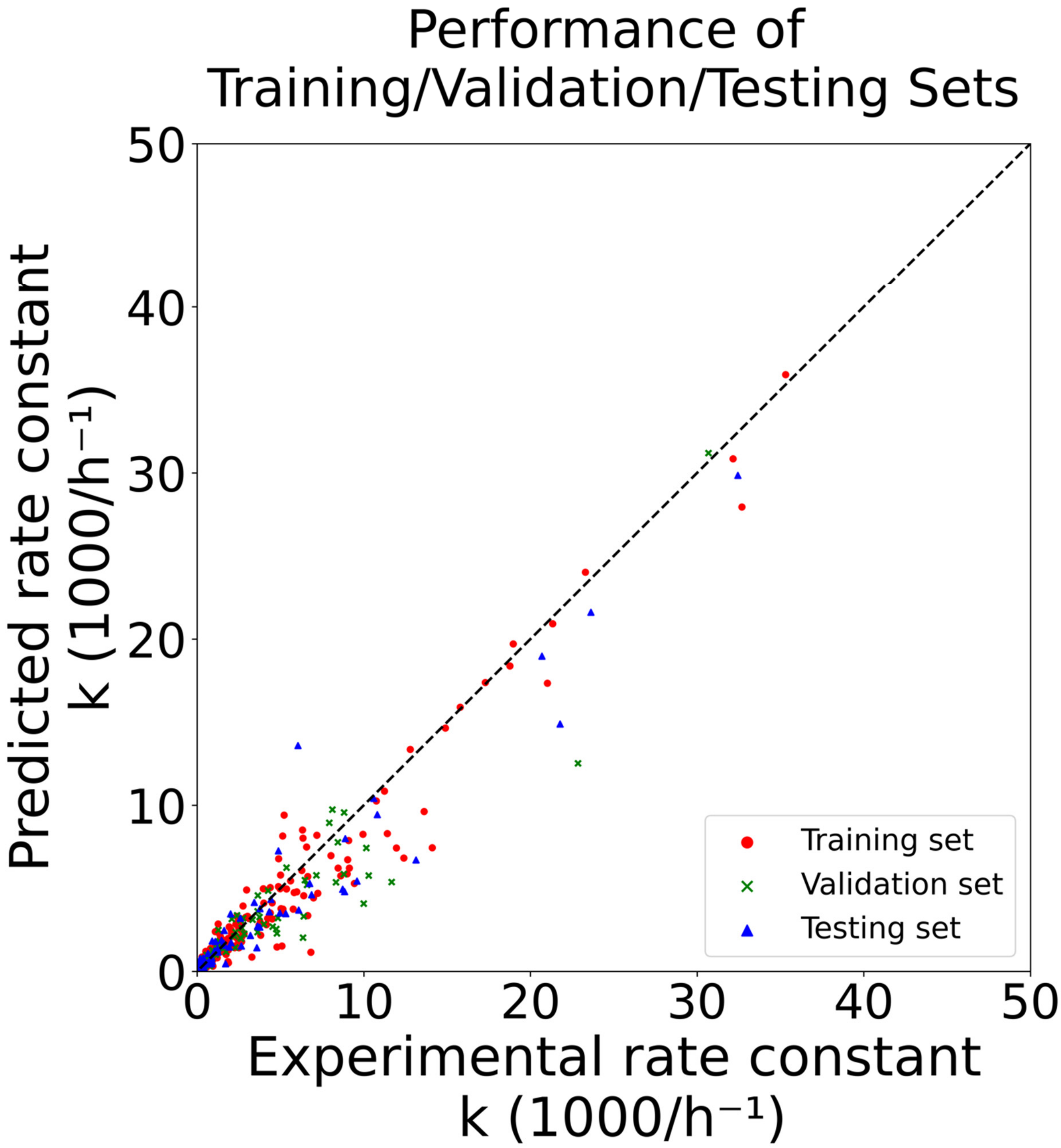

Figure S40. Parity plot of predicted against experimental k for cross validation set 2

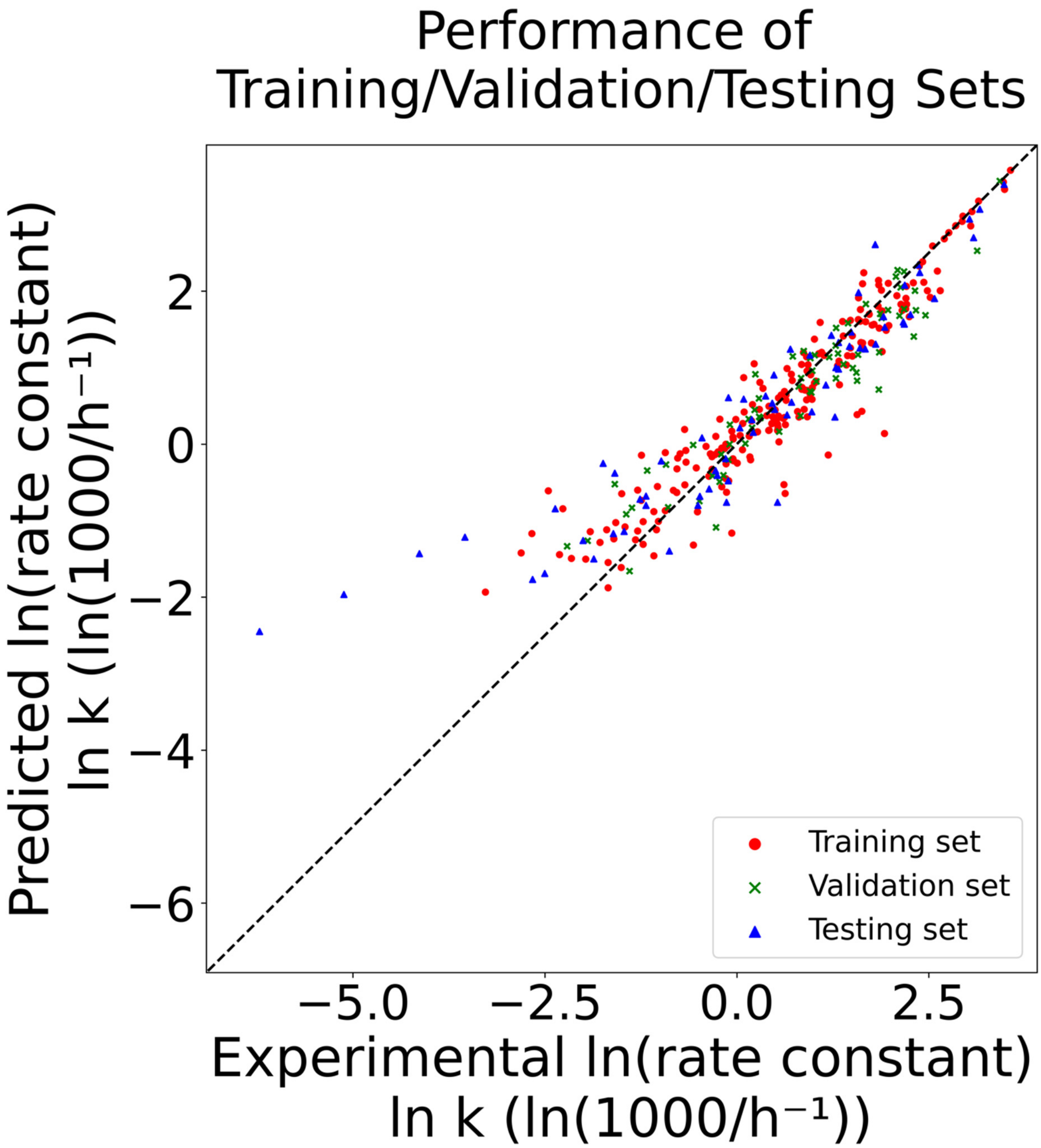

Figure S41. Parity plot of predicted against experimental ln(k) for cross validation set 2

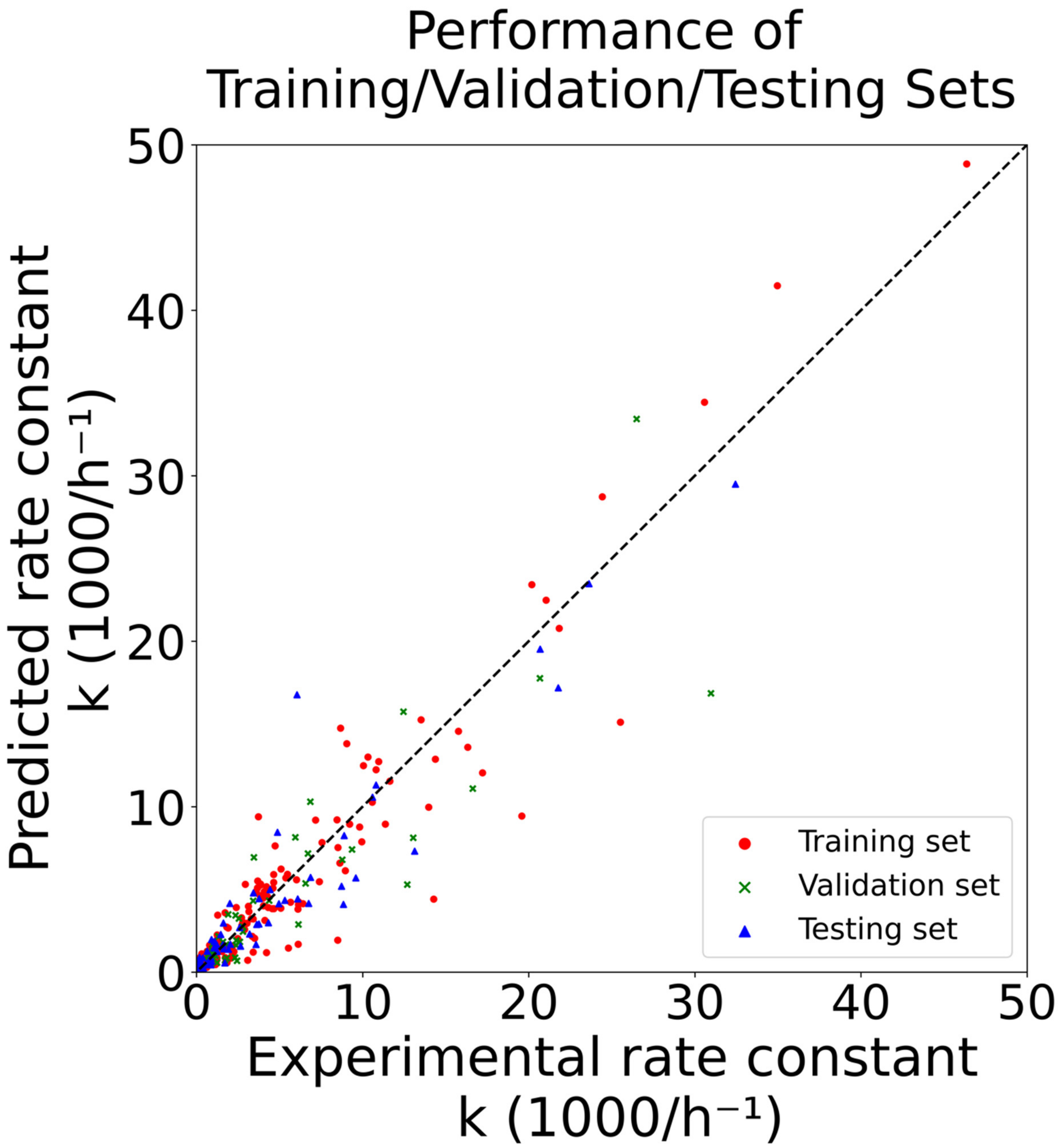

Figure S42. Parity plot of predicted against experimental k for cross validation set 3

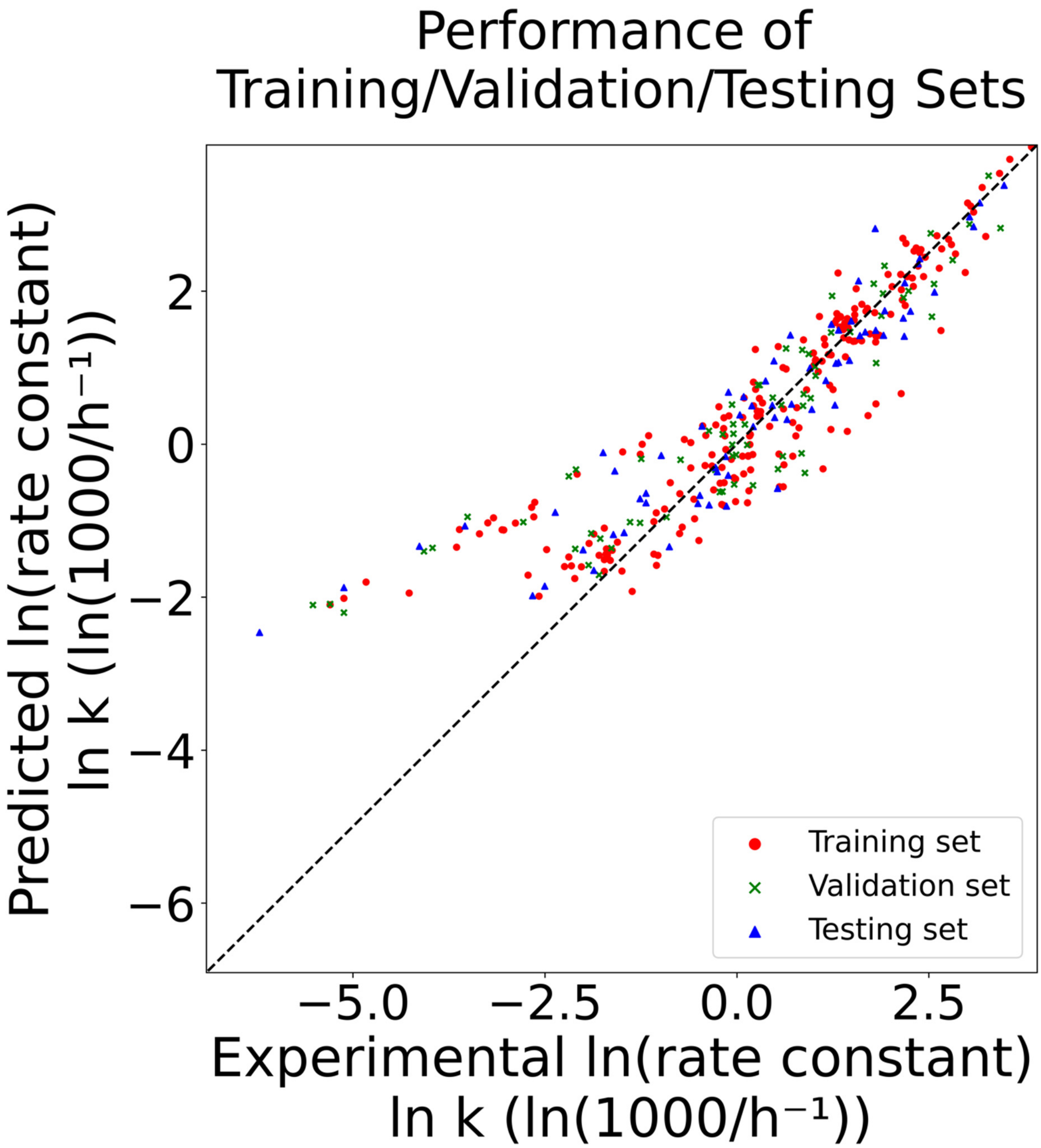

Figure S43. Parity plot of predicted against experimental ln(k) for cross validation set 3

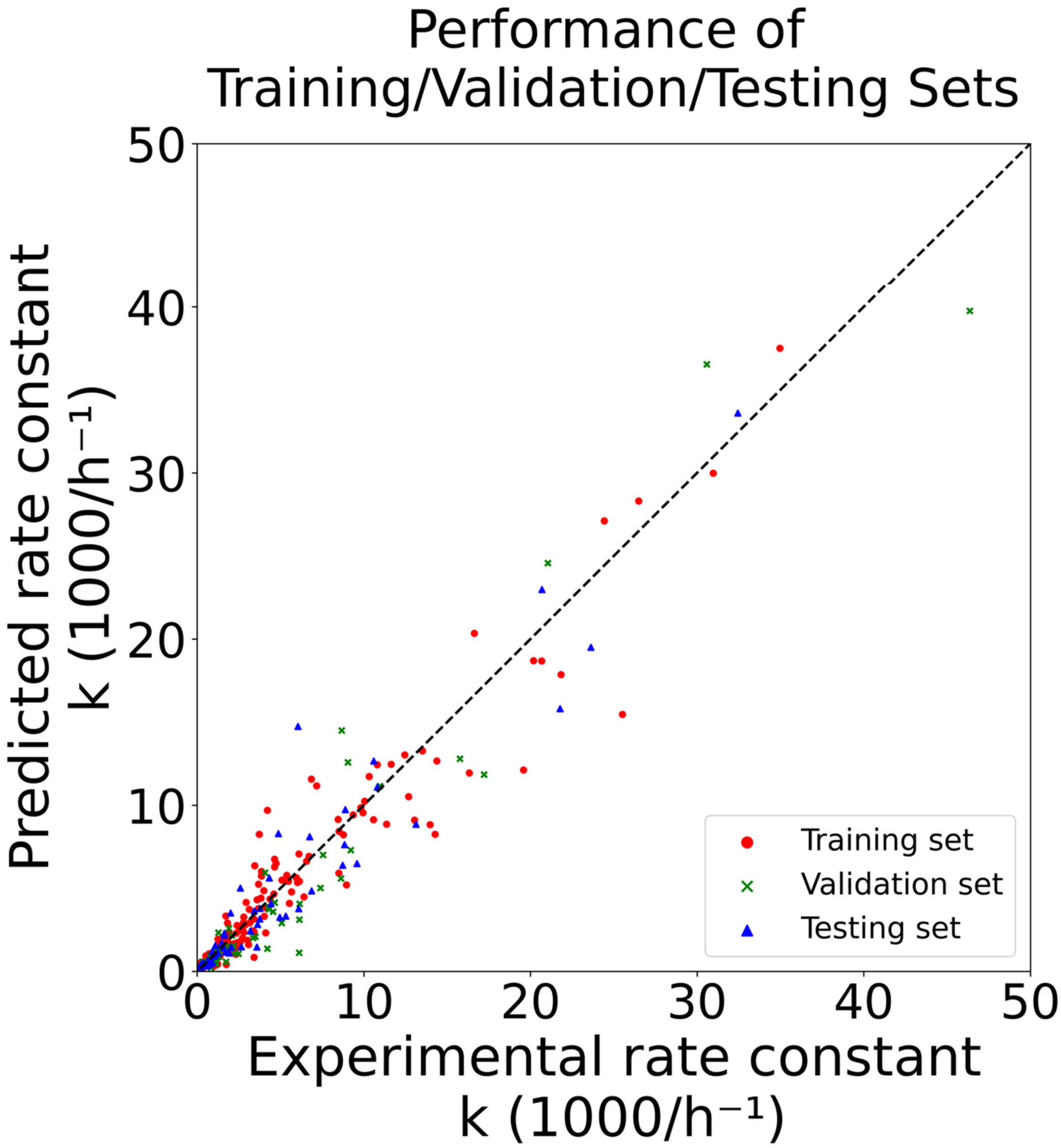

Figure S44. Parity plot of predicted against experimental k for cross validation set 4

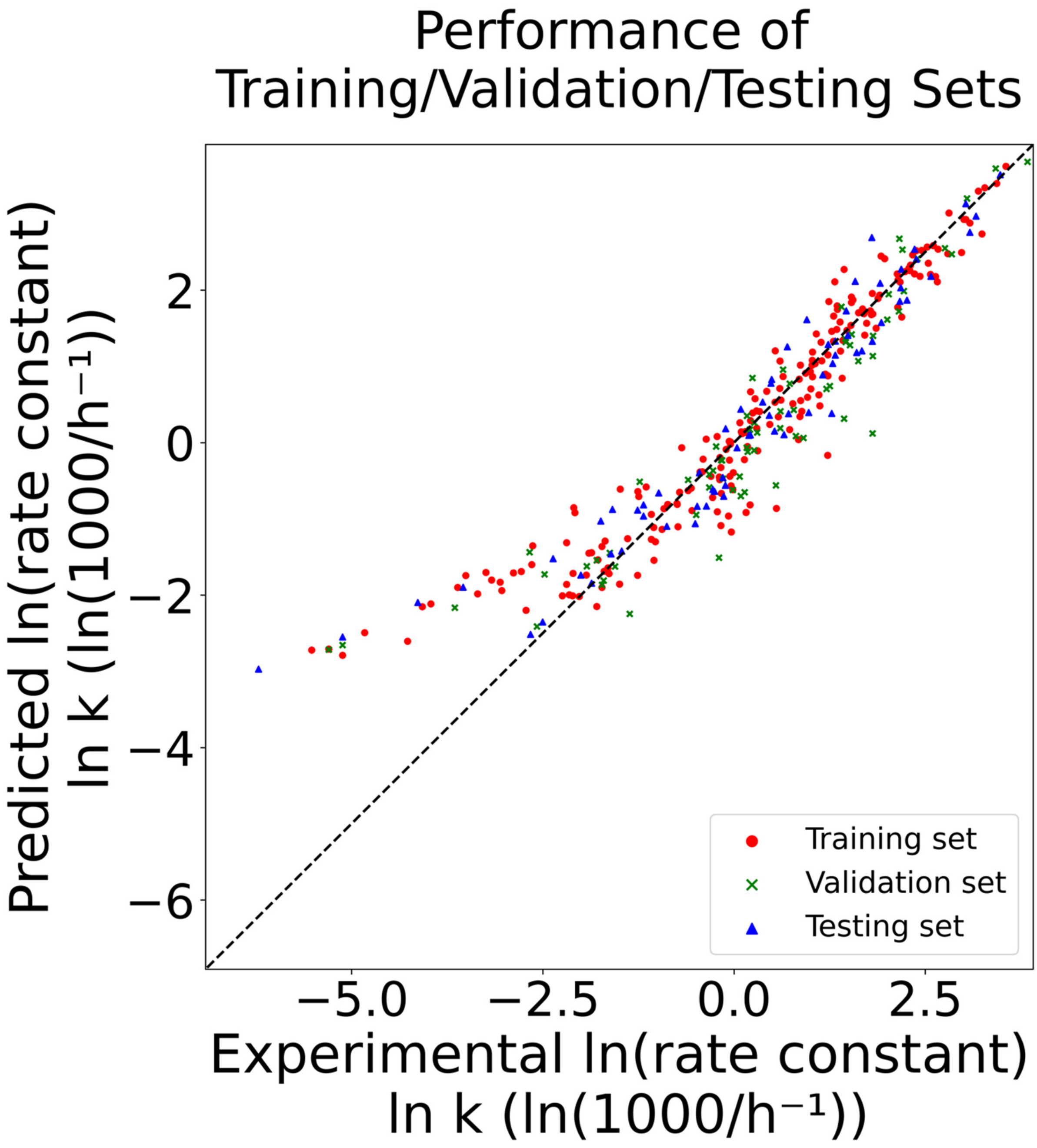

Figure S45. Parity plot of predicted against experimental ln(k) for cross validation set 4

Table S1: Results of Cross-Validation in ln(k)

Dataset	Cross-Validation Set Performance (R^2)			
	Set 1	Set 2	Set 3	Set 4 [1]
Training Set	0.88	0.83	0.67	0.82
Validation Set	0.86	0.82	0.40	0.79
Testing Set	0.89	0.54	0.55	0.80

[1] Average of 3 models were used for this set.

5. Additional tables

Table S2: Averaged predicted reaction rate constants (1000 h^{-1})

		Ligands								
		**1**	**2**	**3**	**4**	**5**	**6**	**7**	**8**	**9**
**Reactants**	1	0.3588	1.9786	0.9252	1.5998	0.7205	5.9299	8.1859	11.3698	20.2018
	2	0.2430	1.4863	0.7005	1.1371	0.5470	3.8752	5.2165	8.2405	13.5515
	3	0.1347	0.9689	0.4624	0.6801	0.3626	2.0247	2.6149	5.0790	7.3860
	4	0.0993	0.7797	0.3744	0.5228	0.2943	1.4462	1.8255	3.9653	5.3964
	5	0.0920	0.7387	0.3553	0.4896	0.2794	1.3293	1.6680	3.7282	4.9892
	6	0.0694	0.6051	0.2928	0.3841	0.2308	0.9716	1.1921	2.9681	3.7282
	7	0.0854	0.7007	0.3375	0.4591	0.2656	1.2238	1.5267	3.5099	4.6197
	8	0.0721	0.6215	0.3005	0.3969	0.2368	1.0136	1.2475	3.0607	3.8778
	9	0.1036	0.8034	0.3855	0.5422	0.3029	1.5154	1.9190	4.1036	5.6374
	10	0.1161	0.8719	0.4173	0.5985	0.3276	1.7201	2.1973	4.5037	6.3431
	11	0.0644	0.5741	0.2783	0.3602	0.2194	0.8939	1.0901	2.7944	3.4505
	12	0.0262	0.3087	0.1526	0.1681	0.1211	0.3279	0.3696	1.3661	1.3631
	13	0.0228	0.2806	0.1391	0.1494	0.1106	0.2799	0.3115	1.2225	1.1781
	14	0.0198	0.2548	0.1267	0.1325	0.1008	0.2385	0.2617	1.0927	1.0162
	15	0.0170	0.2303	0.1149	0.1169	0.0915	0.2014	0.2178	0.9712	0.8696
	16	0.0305	0.3420	0.1685	0.1908	0.1336	0.3879	0.4434	1.5383	1.5923
	17	0.0133	0.1951	0.0979	0.0951	0.0781	0.1522	0.1606	0.7999	0.6721
	18	0.0167	0.2273	0.1135	0.1151	0.0904	0.1971	0.2128	0.9567	0.8525
	19	0.0163	0.2235	0.1116	0.1127	0.0890	0.1916	0.2063	0.9381	0.8305
	20	0.0070	0.1271	0.0647	0.0556	0.0519	0.0729	0.0718	0.4831	0.3418

		Ligands							
		**10**	**11**	**12**	**13**	**14**	**15**	**16**	**17**
**Reactants**	1	16.0895	18.0778	15.0432	31.6254	31.6446	27.1619	36.9081	44.2216
	2	10.8273	12.8013	10.8918	21.4155	21.8392	19.4911	26.7643	30.8516
	3	5.9305	7.6047	6.7024	11.8476	12.4551	11.8231	16.5034	17.8992
	4	4.3462	5.8242	5.2284	8.7308	9.3309	9.1579	12.8967	13.5368
	5	4.0211	5.4490	4.9148	8.0889	8.6813	8.5924	12.1271	12.6242
	6	3.0126	4.2578	3.9095	6.0921	6.6428	6.7857	9.6567	9.7458
	7	3.7260	5.1051	4.6260	7.5056	8.0886	8.0726	11.4184	11.7900
	8	3.1323	4.4016	4.0318	6.3296	6.8868	7.0047	9.9570	10.0915
	9	4.5376	6.0424	5.4113	9.1078	9.7113	9.4855	13.3407	14.0686
	10	5.1011	6.6820	5.9409	10.2183	10.8278	10.4460	14.6441	15.6327
	11	2.7904	3.9888	3.6799	5.6507	6.1879	6.3753	9.0930	9.1005
	12	1.1128	1.8340	1.7941	2.2943	2.6512	3.0373	4.4530	4.0188
	13	0.9633	1.6250	1.6048	1.9920	2.3223	2.7067	3.9859	3.5376
	14	0.8323	1.4379	1.4338	1.7262	2.0308	2.4091	3.5638	3.1093

15	0.7134	1.2643	1.2737	1.4845	1.7634	2.1315	3.1683	2.7146
16	1.2978	2.0870	2.0212	2.6674	3.0539	3.4354	5.0130	4.6050
17	0.5530	1.0227	1.0482	1.1569	1.3968	1.7422	2.6106	2.1699
18	0.6995	1.2438	1.2547	1.4562	1.7320	2.0985	3.1213	2.6681
19	0.6817	1.2173	1.2301	1.4199	1.6915	2.0560	3.0606	2.6081
20	0.2834	0.5886	0.6317	0.6018	0.7595	1.0310	1.5786	1.2094

Table S3. List of Hammett constants used [1]

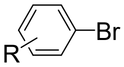	σ_m	σ_p
$-NO_2$[a]	-	0.78
-CN[a]	-	0.7
-SOMe	0.52	0.49
-COMe	0.36	0.47
$-CO_2Et$	0.37	0.45
$-CF_3$	0.46	0.53
-F	0.34	0.06
-H	0	0
-Me	-0.06	-0.14
-OMe	0.11	-0.28
-*t*Bu [a]	-	-0.15
$-NMe_2$	-0.15	-0.83

[a] The *meta*-substitued isomers of these arylbromides were not in the dataset, thus σ_m of these entries were not included.

References

1. Perrin, D. D.; Dempsey B., Serjeant E. P. *pKa prediction for organic acids and bases*; Publisher: Springer, New York, United States, 1981, pp. 109-126
